# IMU-based gait analysis methods: a systematic review of techniques for different body locations

**DOI:** 10.3389/fspor.2026.1854615

**Published:** 2026-07-31

**Authors:** Leqin Chen, Ruiwu Guo, Ruiwen Guo, Yuting Chen, Yinfeng Wang, Shihao Zhang, Qingtong Zhang

**Affiliations:** 1 Shanxi Normal University, Taiyuan, Shanxi, China; 2 Anyang Normal University, Anyang, Henan, China; 3 Jilin Sport University, Changchun, Jilin, China

**Keywords:** algorithm, algorithm comparison, gait analysis, inertial measurement unit (IMU), screening method, sensor placement

## Abstract

**Background:**

Gait analysis is a crucial tool for evaluating human motor function, with its applications expanding across clinical and health-monitoring domains. The growth of the aging global population and an increasing emphasis on sports health have positioned gait abnormalities as significant biomarkers for assessing fall risk and neurological disorders, including Parkinson's disease. Compared with conventional optical systems, inertial measurement units (IMUs) provide a high-precision, cost-effective, and portable alternative, thereby facilitating the practical capture of gait data in real-world contexts.

**Objective:**

This review aims to deliver a comprehensive guide for selecting the most suitable IMU-based gait analysis methodologies, tailored to various application scenarios. Unlike existing reviews, which primarily focus on specific algorithms or populations, this study uniquely synthesizes current IMU-based methods from a sensor-placement perspective. Furthermore, we propose a practical decision framework to guide researchers and clinicians in selecting the optimal sensor locations and algorithms tailored to specific application scenarios and computational constraints.

**Method:**

A systematic search was conducted across the China National Knowledge Infrastructure, Wanfang, PubMed, and Web of Science databases from January 2019 to December 2025 using relevant English and Chinese keywords related to IMUs and gait analysis. To ensure methodological completeness in this mature field, foundational studies published before 2019 were additionally included through targeted supplementary retrieval. The retrieved studies underwent a systematic review.

**Results and conclusions:**

This paper provides a comprehensive comparison of IMU-based gait analysis methods across various sensor placements on the body (e.g., foot, leg, chest). The findings reveal substantial differences in algorithm complexity, accuracy, and suitability across distinct populations and scenarios. This study establishes an evidence-based framework for identifying optimal gait analysis solutions to address diverse application needs.

## 1 Introduction

Gait analysis in the fields of sports science and biomechanics holds significant value for clinical diagnosis, the optimization of sports performance, and the development of intelligent equipment. Inertial sensors exhibit considerable potential to support clinical decision-making for gait-related diseases. Objective spatio-temporal gait analysis typically requires precise sensor placement on the body surface to evaluate the sensor data within the body coordinate system (1). In addition, spatio-temporal gait parameters serve as crucial biomarkers for monitoring gait disorders and developing rehabilitation systems (2).

For example, in the diagnosis and treatment of Parkinson's disease, the characteristic gait changes exhibited by patients provide a critical basis for disease assessment (3). During the rehabilitation stage, precise gait analysis helps formulate individualized rehabilitation plans (4). Moreover, IMUs can capture movement data with high-fidelity in real-world sports scenarios (5). Although traditional optical motion capture systems are considered the “gold standard” for gait analysis (6–10), their high cost and stringent requirements for site and lighting conditions severely limit their applicability in everyday scenarios. However, IMUs can effectively address these significant limitations. This field typically focuses on using one or a few sensors to collect motion data (11).

Advances in technology have made inertial measurement units (IMUs) highly feasible and ubiquitous tool for gait analysis. A proliferation of research has emerged in recent years, particularly since 2019. Marked by the establishment of large-scale international digital mobility consortia such as the Mobilise-D of the European Union, IMU-based gait analysis has officially undergone a paradigm shift from controlled laboratory environments to continuous “free-living” settings. This revolution has achieved critical milestones in recent years. As reported in the 2024 official outcomes of the Mobilise-D consortium, collaborative efforts between academia and the clinical sector are actively dismantling barriers to the widespread adoption of digital mobility assessments by establishing “standardized, scalable, and regulatory-approved generic solutions.” Driven by this overarching trend, numerous high-quality systematic reviews published between 2019 and 2025 have extensively explored IMU applications. These encompass the assessment and rehabilitation of Parkinson's disease (12, 13), the monitoring of dementia (14) and broader neurological populations (15), the assessment of fall risk (18, 19) in older adults (16) or neurotrauma patients (17), the analysis of sarcopenia (20), the application of sports medicine (21), and the assessment of routine walking in healthy populations (22).

Although existing disease-specific reviews (12–22) offer invaluable clinical insights, from the perspective of underlying algorithmic architecture, the anatomical placement of the IMU remains the fundamental determinant of the data-processing pipeline. Consequently, serving as a critical supplement to clinically oriented literature, this review innovatively categorizes and systematically analyzes gait analysis algorithms based on their anatomical sensor placement. By comprehensively elucidating the theoretical principles, comparative advantages and limitations, and applicable scenarios of various methodologies, this review aims to provide a clear, versatile decision framework and algorithm selection guide for researchers, thereby further facilitating the cross-disciplinary application of IMU technology.

The necessity for such a universal algorithmic framework is particularly pronounced in real-world environments. In unconstrained daily life, individuals routinely adjust their gait patterns in response to dynamic environmental conditions. Therefore, IMU-based gait analysis algorithms must remain robust enough to provide reliable measurements despite these spontaneous gait alterations (23). Consequently, prior to calculating specific gait parameters (e.g., stride length, cadence, and gait cycle duration), it is imperative to use algorithmic pipelines specifically optimized for diverse sensor placements to accurately filter and classify raw IMU data and effectively distinguish among various complex gait patterns.

## 2 Methods

### 2.1 Retrieval strategy

**Figure 1:**
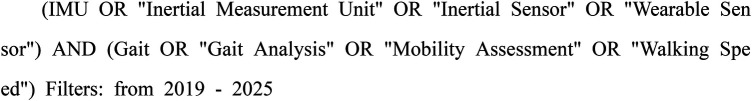
Core PubMed search strategy diagram.

Two independently trained reviewers systematically conducted the literature search. The search encompassed literature published from January 2019 to December 2025. The following electronic databases were searched: the China National Knowledge Infrastructure (CNKI), Wanfang Database, PubMed, and the Web of Science. The search strategy utilized specific terms in both English and Chinese. English search terms included “IMU,” “Inertial Measurement Unit,” “Wearable Sensor,” “Gait Analysis,” and “Gait.” Corresponding Chinese search terms were “inertial measurement unit” and “gait analysis.” The retrieved literature types included primary research articles and review articles. A detailed search strategy was formulated for each database. The search strategy for the PubMed database is provided as an example in Figure 1.

### 2.2 Literature screening and inclusion and exclusion criteria

This systematic review was conducted and reported in strict accordance with the Preferred Reporting Items for Systematic Reviews and Meta-Analyses (PRISMA) 2020 guidelines. The inclusion criteria are rigorously defined as follows: (1) focused on human gait analysis; (2) utilized IMUs as the primary assessment tool; (3) proposed novel algorithms or technically evaluating existing computational methods; (4) validated results against an independent reference standard (e.g., optical motion capture, force plates); (5) reported quantitative gait parameters (e.g., spatio-temporal metrics, event detection accuracy); and (6) were published as original peer-reviewed research. The exclusion criteria included: involving non-human participants; unavailable full texts (e.g., abstracts, editorials); insufficient methodological details or missing key data; purely clinical applications lacking algorithmic validation; secondary literature such as systematic reviews or meta-analyses (to prevent the double-counting of evidence and the mixing of primary validation data with secondary interpretations); and findings that had been fully superseded by updated, more reliable evidence. While review articles were strictly excluded from the final quantitative and qualitative synthesis, they were consulted exclusively for background contextualization and manual cross-checking of references during the initial scoping phase.

To ensure rigorous screening consistency and mitigate selection bias, a pilot screening of 50 randomly selected records was conducted prior to the formal evaluation, achieving an initial inter-reviewer agreement rate exceeding 90%. Following this calibration phase, two reviewers independently screened the literature. A total of 4,039 records were retrieved. After removing 1,669 duplicate records, 2,370 records remained. The two reviewers independently screened the titles and abstracts these 2,370 records, excluding 1,468 records in total. The excluded records included 132 with unclear or ambiguous results, 86 with incomplete or insufficient data, 256 involving non-human participants, and 994 irrelevant to the topic of IMU-based gait analysis.

**Figure 2:**
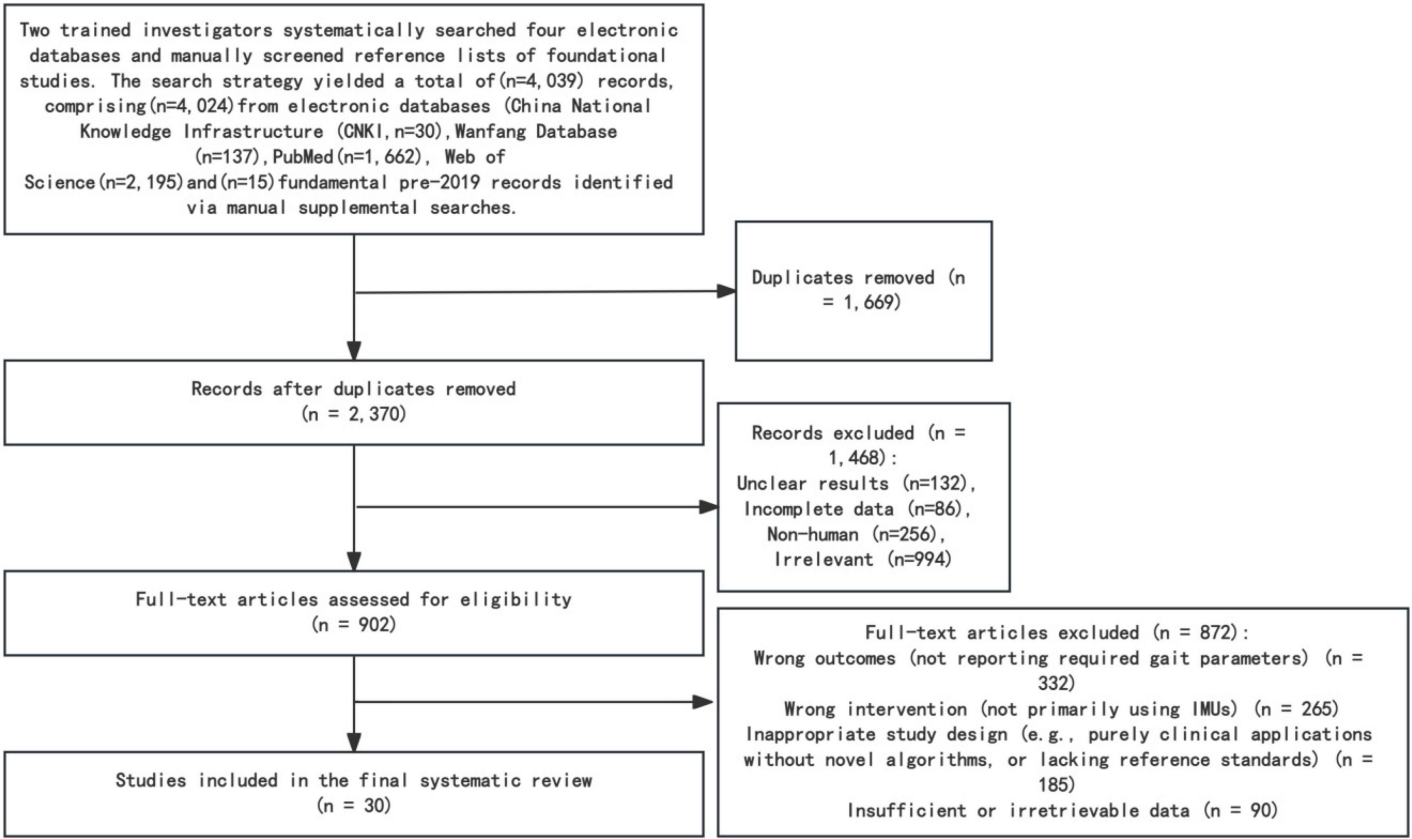
Flowchart of article screening.

After the initial screening, 902 records remained and proceeded to the full-text assessment stage. During the full-text review, an additional 872 studies were excluded for specific methodological reasons. The detailed exclusion breakdown is as follows: studies not reporting relevant quantitative gait parameters (*n* = 332); studies not utilizing IMUs as the primary assessment tool (*n* = 265); review articles or inappropriate study designs, including purely clinical applications without proposing novel algorithms, studies lacking reference standards, and secondary literature (*n* = 185); and studies with insufficient or irretrievable core data (*n* = 90). Any discrepancies between reviewers were resolved through detailed discussion or arbitration by a third independent senior researcher to reach a final consensus. Ultimately, 30 studies satisfied all predefined inclusion criteria and were included in the final systematic review. The detailed literature screening process is illustrated in the PRISMA flowchart in Figure 2.

**Figure 3:**
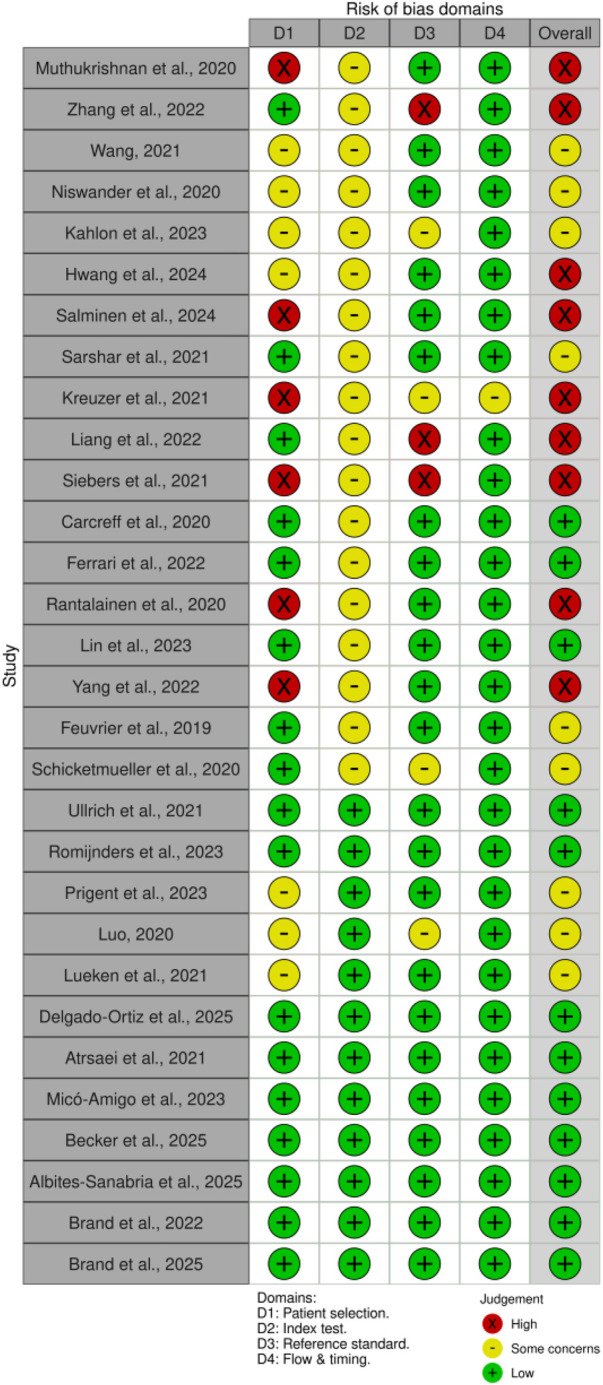
Quality assessment and risk of bias summary of the included studies using the QUADAS-2 tool.

### 2.3 Quality assessment

Following the systematic screening process, 30 studies were included in the final review. The methodological quality and risk of bias of these included studies were independently evaluated by two reviewers using the Quality Assessment of Diagnostic Accuracy Studies 2 (QUADAS-2) tool. The assessment encompassed four key domains: patient selection, index test, reference standard, and flow and timing. To ensure rigorous screening consistency and mitigate selection bias, a pilot screening of 50 randomly selected records was conducted prior to the formal evaluation, achieving an initial inter-reviewer agreement rate exceeding 90%. Throughout the formal screening and quality assessment stages, any minor discrepancies in study selection or QUADAS-2 domain judgments were resolved through detailed discussion or arbitration by a third independent senior researcher, thereby strictly safeguarding the objectivity and reproducibility of the inclusion process.

## 3 Results

### 3.1 Flowchart of literature screening

Flowchart of article screening is illustrated in Figure 2.

### 3.2 Quality assessment of included studies

Quality assessment of included studies is illustrated in Figure 3.

### 3.3 Review of pre-2019 foundational algorithms

As illustrated in Table 1, selecting an appropriate gait detection algorithm necessitates a tradeoff among computational resource consumption, real-world reliability, and convenience. Traditional methods are computationally efficient and theoretically intuitive, making them highly suitable for low-power wearable devices; however, they often fall short when addressing the complex and highly variable pathological gaits encountered outside laboratory settings. In contrast, machine learning and deep learning models demonstrate superior accuracy and robustness to noise during unconstrained daily walking. Nevertheless, this enhanced performance comes at the cost of substantial computational demands and a lack of clinical transparency (i.e., the “black-box” problem).

**Table 1:** Foundational and classical IMU-based gait analysis algorithms (pre-2019).

Technological paradigm	Author and year	Algorithm/method	Application scope and core contributions
Gait event detection	Catalfamo et al., 2010	Single-shank gyroscope peak detection algorithm (24)	Application scope: Detection of initial contact (IC) and foot off (FO) events across complex terrains (level ground and inclines)
			Core contribution: Demonstrated that a single shank-mounted gyroscope can robustly extract gait phases in unconstrained outdoor environments, significantly advancing the clinical usability of wearable sensors
Spatial parameter estimation and drift suppression	Foxlin, 2005	Zero-velocity update (ZUPT) and Kalman filter navigation (25)	Application scope: Infrastructure-free 3D pedestrian tracking using shoe-mounted IMUs
			Core contribution: Systematized the application of stance-phase zero-velocity constraints to effectively suppress the double-integration drift of accelerometers. This established an indispensable theoretical framework for all subsequent estimates of foot trajectory and stride length
Spatial parameter estimation and drift suppression	Sabatini et al., 2005	Foot inertial sensing-based strap-down integration and phase segmentation (26)	Application scope: High-precision estimation of walking speed, stride length, and ground incline using a foot-mounted bi-axial accelerometer and single-axis gyroscope
			Core contribution: Proposed an efficient gait phase segmentation procedure that strictly limits integration drift by precisely defining integration time intervals, laying the absolute foundation for high-accuracy foot-based gait assessment
Advanced kinematic modeling and drift reduction	Fasel et al., 2017	Joint-constrained acceleration mapping for orientation drift reduction (27)	Application scope: 3D segment orientation and joint angle estimation during highly dynamic movements (e.g., alpine skiing) where typical quasi-static (zero-velocity) phases are absent
			Core contribution: Exploited mutual information from adjacent sensor units by mapping 3D accelerations to the connecting joint. This novel biomechanical constraint successfully reduced integration drift in highly dynamic scenarios, overcoming the fundamental limitations of traditional single-sensor ZUPT methods
Gait event detection	Jasiewicz et al., 2006	Linear accelerometer and angular velocity threshold detection (28)	Application scope: Detection of initial contact (IC) and end contact (EC) in both normal and spinal-cord-injured patients
			Core contribution: Demonstrated that foot linear accelerations or angular velocity methods are as accurate as standard pressure-sensitive foot switches for estimating IC and EC timing in normal gait patterns
Gait event detection	Kotiadis et al., 2010	Inertial sensor-based threshold techniques (29)	Application scope: Gait phase detection to replace heel switches for triggering drop foot stimulators
			Core contribution: Developed low computational load algorithms using accelerometers and gyroscopes. Showed 100% detection on flat and rough terrain while effectively ignoring non-gait events like weight shifts
Spatial parameter estimation	Aminian et al., 2002	Wavelet analysis and mechanical model (30)	Application scope: Long-term ambulatory estimation of spatio-temporal parameters using miniature lower-limb gyroscopes
			Core contribution: Proposed computing stride length and velocity by integrating angular velocity based on the medio-lateral rotation of lower limbs, enabling longitudinal monitoring without provoking patient discomfort
Spatial parameter estimation	Zijlstra and Hof, 2003	Center of mass trajectory model (31)	Application scope: Assessment of spatio-temporal gait parameters from trunk accelerations
			Core contribution: Developed algorithms to determine step length, walking speed, and step duration from lower trunk accelerations based on model predictions of the body's center of mass trajectory during walking
Spatial parameter estimation and drift suppression	Sabatini, 2005	Quaternion-based strap-down integration (32)	Application scope: Orientation and positioning estimates using inertial sensing
			Core contribution: Exploited the cyclical nature of gait by double integrating gravity-compensated accelerometer signals during the swing phase. Developed an interpolation technique to improve accuracy by accounting for sensor bias and scale factor drifts
Spatial parameter estimation	Li et al., 2010	Inverted pendulum-like direct integration (33)	Application scope: Estimating walking speed using a shank-mounted inertial measurement unit
			Core contribution: Utilized the inverted pendulum-like behavior of the stance leg to divide stride cycles and estimate initial conditions for direct integration, providing a reliable alternative to foot-mounted sensors
Drift suppression	Peruzzi et al., 2011	Zero-velocity assumption validation (34)	Application scope: Estimation of stride length via double integration in level walking
			Core contribution: Investigated the hypothesis that sensor velocity is zero during stance. Validated that this zero-velocity assumption is acceptable primarily when the sensor is attached to the foot (e.g., calcaneus) rather than the shank
Spatial parameter estimation	Schepers et al., 2010	Multi-stride integration path estimation (35)	Application scope: Ambulatory estimation of lateral foot placement (LFP) and stride length (SL)
			Core contribution: Significantly reduced integration drift by defining LFP and SL with respect to an average walking path using a predefined optimal number of consecutive strides
Foot trajectory and clearance	Mariani et al., 2010	Optimized fusion and de-drifted integration (36)	Application scope: Assessment of 3D foot kinematics (stride length, velocity, foot clearance, turning angle) in young and elderly participants
			Core contribution: Validated a wearable wireless system that computes spatial parameters at each gait cycle, successfully observing gait performance differences (particularly foot clearance) between age groups outside the lab environment
Foot clearance validation	Dadashi et al., 2013	Shoe-worn clearance parameterization (37)	Application scope: Providing normative values for gait and foot clearance parameters
			Core contribution: Extracted parameters using validated algorithms from a large-population sample (>1,400 older adults) to investigate differences by gender and age, offering a unique clinical reference resource for understanding falls
Algorithm benchmarking	Hannink et al., 2017	Estimation pipeline benchmarking (38)	Application scope: Reconstruction of foot trajectories for mobile gait analysis
			Core contribution: Addressed the lack of benchmark datasets by implementing and comparing three orientation estimation and three double integration schemes on a shared dataset of 735 strides, identifying the most suitable processing pipeline

More importantly, algorithmic performance cannot be evaluated in isolation. It is fundamentally dependent on the characteristics of the raw input signal, which are directly determined by the anatomical placement of the IMU sensor. During walking, distinct body segments (e.g., the trunk, lower limbs, or wrists) generate kinematic trajectories and signal-to-noise ratios that differ significantly. Consequently, the specific sensor location fundamentally dictates the type of algorithmic pipeline required for data processing. Driven by this practical rationale, the subsequent sections provide a comprehensive review of current IMU-based gait analysis methods, categorizing them by sensor mounting location.

### 3.4 Technical landscape and computational characteristics of IMU-based algorithms

To systematically evaluate the algorithmic architectures, a standardized rubric was defined to classify computational complexity. Because most clinical validation studies do not explicitly report Big-O notation or memory footprints, the computational categories (low, moderate, high) and theoretical complexities were inferred from the authors. This inference was strictly based on the core mathematical paradigms and data-processing pipelines described in the original methodologies. The classification rubric for time consumption and memory usage is defined as follows:

1. Low complexity: Algorithms operating in constant O(1) or linear O(N) time (e.g., heuristic thresholds, standard numerical integration, complementary filters). These algorithms feature minimal memory usage and are suitable for real-time execution on ultra-low-power embedded microcontrollers.
2. Moderate complexity: Algorithms scaling at O(N log N) or requiring multiscale operations (e.g., continuous wavelet transform, traditional machine learning classifiers, PCA). These algorithms impose moderate memory and processing demands and typically require standard edge-computing microprocessors.
3. High complexity: Algorithms characterized by polynomial or quadratic time complexities, such as O(L × H^2^), primarily representing deep learning architectures [e.g., Convolutional neural networks (CNNs), LSTMs]. These algorithms require significant RAM (high memory) and advanced processing capabilities for model inference, making them challenging for standard wearable embedded systems.

### 3.5 Summary of key insights from the technical landscape

Several observations emerge from the computational and algorithmic characterization presented in Table 2. First, there exists a clear tradeoff between algorithmic complexity and clinical interpretability: traditional rule-based methods [e.g., cumulative shank angular velocity (CSAV) (39), Martin and Salaun algorithm (61)] offer low computational cost and transparent decision logic, making them suitable for real-time embedded applications, whereas deep learning approaches (44, 45, 70, 80) achieve superior accuracy and noise robustness at the expense of computational intensity and lack of biomechanical transparency. Second, the choice of sensor placement is non-randomly associated with algorithmic architecture: foot-mounted and lower-limb configurations more frequently employ both traditional and deep learning methods for event detection and phase estimation, while lower-back and wrist placements increasingly leverage standardized pipelines and self-supervised frameworks designed for longitudinal, free-living monitoring (74, 76, 77, 79, 82). Third, the computational complexity classifications reported in Table 2 were derived by inference from the author based on the described architectures, as primary studies rarely report runtime or memory metrics; this gap underscores the need for future work to systematically document computational performance to facilitate fair cross-algorithm comparisons. Collectively, these observations suggest that algorithm selection should be guided not only by accuracy but also by the specific deployment context, hardware constraints, and clinical interpretability requirements of the intended application.

**Table 2:** Technical landscape, computational complexity, and algorithmic characteristics of IMU-based gait analysis methods.

Algorithm	Core technical approach	Input signals	Computational complexity (single-step time consumption/memory usage/computational complexity order)	Algorithmic pros and cons
Parkinson's disease standardized gait test detection algorithm based on foot IMU (Ullrich et al.) (68)	Template matching and gait sequence decomposition	Multi-axis IMU	Moderate; low; O(N × m)	Pros: Automated detection of unsupervised standardized gait tests using robust sDTW template matching without manual annotations
				Cons: Requires fixed template definitions; susceptible to overlapping matches and non-compliant test executions
The Martin and Salaun algorithm (Feuvrier et al.) (61)	Complementary filtering attitude fusion	Multi-axis IMU	Low; Low; O(1)	Pros: Extremely low computational footprint; high precision in angular estimation
				Cons: Performance during non-straight-line kinematic trajectories remains unstable
Unrestricted step length detection method for staircase climbing based on IMU (Hannah et al.) (51)	Multi-stage heuristic thresholding (peak and zero-crossing)	Multi-axis IMU	Low; Low; O(N)	Pros: High adaptability using dynamically bounded search windows
				Cons: Susceptible to complex signal noise in non-standard scenarios
Algorithm for detecting the optimal kinematic parameter signals of the legs (Kahlon et al.) (10)	Similarity analysis and Poincaré plot analysis	Multi-axis IMU	Moderate; Low; O(N)	Pros: Effectively identifies key non-linear dynamic signals
				Cons: Requires specialized signal pre-processing procedures
The KF+ model algorithm (Lueken et al.) (73)	State estimation and kinematic modeling (Kalman filter + inverted pendulum)	Multi-axis IMU	Low; Low; O(N)	Pros: Highly automated vertical displacement estimation
				Cons: Requires manual input of leg length; significant error increases at very low speeds
The SDI-step algorithm (Muthukrishnan et al.) (2)	Sensor data integration (velocity/position drift correction)	Multi-axis IMU	Low; Low; O(N)	Pros: Computationally efficient drift correction for spatial parameters
				Cons: Generalizability of drift constraints across varying cadences requires further validation
Gait variability assessment algorithm for healthy older adults (Rantalainen et al.) (55)	Non-linear dynamics (refined composite multiscale entropy)	Multi-axis IMU	Moderate; Low; O(N * *τ*)	Pros: Deeply quantifies complex signal variability and environmental adaptability
				Cons: Reliability of entropy metrics in highly noisy signal environments is questionable
Weighted average ensemble classification model (Lin et al.) (57)	Supervised machine learning (SVM/random forest)	Multi-dimensional features	Moderate; Low; O(N)	Pros: Achieves high accuracy in multidimensional kinematic differential classification
				Cons: Feature engineering burden is high; processing multi-node data increases operational complexity
Cumulative shank angular velocity (CSAV) algorithm (Mikko et al.) (39)	Direct signal integration (cumulative shank angular velocity)	1D angular velocity	Low; Low; O(N)	Pros: Extremely high algorithmic stability with negligible computational cost
				Cons: Sub-optimal detection rate for subtle kinematic events like heel rise
Long short-term memory (LSTM)-based algorithm for gait phase estimation using leg-mounted IMUs (LSTM-LIGPE) (Sarshar et al.) (44)	Supervised deep learning (long short-term memory network)	Multi-axis IMU + Rotation matrix	High; High; O(L × H^²^)	Pros: Fully automated end-to-end architecture bypassing manual feature engineering
				Cons: High memory footprint; heavily reliant on massive, high-quality annotated training data
Orientation-invariant spatio-temporal gait analysis algorithm using foot-worn IMUs (OI-STGA) (Guimarães et al.) (1)	Quaternion complementary filter (Euston/Madgwick) and direct/reverse integration	Multi-axis IMU	Low; Low; O(N)	Pros: Achieves orientation-invariant robustness using computationally lightweight filters without complex Kalman matrix inversions
				Cons: Direct and reverse integration requires complete stride data, hindering strict real-time execution
Machine learning-based leg-IMU abnormal gait classification algorithm (ML-LIGCA) (Hwang et al.) (11)	Machine learning classification inference	Multi-axis IMU features	Moderate; Low; O(N)	Pros: High classification accuracy for specific localized kinematic impairments
				Cons: Algorithmic performance is heavily constrained by environmental noise
Real-time lower-limb kinematic estimation via MLR and Kalman filter (Wang) (8)	Multiple linear regression (MLR) + Kalman filter fusion and SVM	Multi-axis IMU (bilateral shanks)	Moderate; Low; O(N)	Pros: Accurately extracts real-time multi-joint kinematics (knee, hip, thigh) using only two shank sensors; ZVS detection is highly adaptable to pathological gaits
				Cons: Kinematic modeling is strictly restricted to the sagittal plane; MLR model accuracy relies heavily on the specific training dataset
Self-adaptive algorithm for detecting key contact points during running, based on foot-worn IMUs (Yang et al.) (59)	Adaptive dual-signal thresholding (Accel + Gyro)	Multi-axis IMU	Low; Low; O(N)	Pros: Rapid detection with real-time automatic threshold adjustment
				Cons: Algorithmic logic is highly sensitive to variations in foot strike patterns
Gait event detection algorithm for robot-assisted gait training in stroke patients (Schicketmueller et al.) (64)	Finite state machine (FSM) and angular velocity conditions	1D angular velocity	Low; Low; O(N)	Pros: Extremely low latency state transition suitable for real-time hardware triggers.
				Cons: Narrow kinematic scope; requires optimization for complex non-linear movements.
Foot-IMU-based walking bout detection in real-life settings (Prigent et al.) (71)	Continuous wavelet transform (CWT) and Hilbert envelope	Multi-axis IMU	Moderate; Low; O(N log N)	Pros: Robust signal peak enhancement independent of sensor orientation
				Cons: Algorithm performance relies heavily on the selection of threshold strategies
Foot-IMU-based deep learning gait event detection in mobility-limiting diseases (Romijnders et al.) (70)	Deep learning model forward inference	Multi-axis IMU	High; High; O(L × H^2^)	Pros: High robustness across heterogeneous kinematic patterns without subject-specific calibration
				Cons: “Black-box” architecture lacks direct biomechanical interpretability
Mobilise-D COPD gait analysis algorithm using a waist-worn IMU (Delgado-Ortiz et al.) (74)	Standardized thresholding and data aggregation	Multi-axis IMU	Low; Low; O(N)	Pros: Automated and standardized extraction of 15 comprehensive digital mobility outcomes
				Cons: Algorithmic logic may fail to capture meaningful impairments during ultra-short walking bouts
Multi-site IMU gait assessment in children with CP (Carcreff et al.) (52)	PCA-based axis alignment and double pendulum model	Multi-axis IMU	:Moderate; Low; O(N log N)	Pros: PCA alignment significantly reduces signal errors caused by sensor misplacement
				Cons: The double pendulum mathematical model relies on precise manual measurements of leg segments
CNN-based wrist gait detection algorithm (Brand et al.) (80)	Convolutional neural network (CNN) inference	Multi-axis IMU	High; High; O(L × H^²^)	Pros: Effectively filters out massive non-locomotion noise from complex upper-limb movements
				Cons: High computational resource demands; requires extensive dataset diversity
Self-supervised deep learning for wrist-worn sensors (Brand et al., 2025) (82)	Self-supervised learning (SSL) using a ResNet backbone, pre-trained on massive unlabeled datasets and fine-tuned for regression-based spatial–temporal gait metric estimation	Single-node IMU (non-dominant wrist measuring 3D acceleration)	High; High; O(W × C × N)	Pros: Maximizes long-term user compliance via wrist placement; SSL drastically reduces the need for extensive labeled data; highly generalizable across diverse pathological cohorts
				Cons: High computational cost for initial training; wrist kinematics are distally decoupled from the lower limbs, making certain parameters harder to predict than with lower-back sensors
IMU gait apraxia assessment algorithm for iNPH (Ferrari et al., 2022) (53)	Multi-sensor synchronization and postural transition thresholding	Multi-axis IMU	Low; Low; O(N)	Pros: Synchronized logic simultaneously captures macro-level transitions and micro-level parameters
				Cons: High signal synchronization overhead increases pre-processing complexity
Lower-back IMU mobility assessment algorithm for fear of falling in PD (Atrsaei et al.) (75)	Logistic regression and decision tree classification	Multi-axis IMU features	Moderate; Low; O(N)	Pros: Efficient multi-domain feature extraction using computationally light classifiers
				Cons: Turning detection logic is prone to misclassifying general non-locomotion rotations
Multi-cohort lower-back real-world gait assessment algorithms (Micó-Amigo et al.) (76)	Frequency-based convolution + CWT + inverted pendulum	Multi-axis IMU	Moderate; Moderate; O(N log N)	Pros: Highly robust fusion pipeline for estimating multiple spatial–temporal parameters
				Cons: Algorithmic performance degrades significantly for irregular slow walkers
Waist-worn slow-gait estimation algorithm for sarcopenia (Mueller et al.) (77)	Short-time Fourier transform (STFT) + linear projection	Multi-axis IMU	Moderate; Moderate; O(N log N)	Pros: Specifically calibrated frequency extraction prevents the typical overestimation of slow gait speeds
				Cons: The linear projection model is over-fitted to low-frequency data and may fail on fast walking
Mobilise-D lower-back computational pipeline for hip fracture recovery (Becker et al.) (78)	Standardized data structuring and walking bout extraction	Multi-axis IMU	Moderate; Moderate; O(N log N)	Pros: Capable of capturing highly granular outcomes across longitudinal recovery phases
				Cons: Relies heavily on predefined minimum wear-time criteria, risking data exclusion
Mobilise-D healthy aging benchmark pipeline (Albites-Sanabria et al.) (79)	Walking bout extraction + generalized additive models (GAMs)	Multi-axis IMU	Moderate; Moderate; O(N log N)	Pros: Incorporates non-linear modeling (GAMs) to capture critical tipping points of mobility decline
				Cons: Normative benchmarks are derived from a specific cohort, requiring cross-cultural validation
ConvLSTM network (Kreuzer and Munz) (45)	Sliding-window 2D-CNN and LSTM networks	Multi-site IMUs (acceleration, rotation, and barometric pressure)	High; High; O(W × N × C)	Pros: Achieves high granularity by differentiating five distinct gait phases without manual threshold tuning; identifies optimal sensor locations via feature activation analysis
				Cons: Misclassifications predominantly occur at micro-transitions between phases due to temporal resolution limits; lacks physiological transparency
LSTM-based synergy modeling (Liang et al.) (46)	Continuous relative phase (CRP) extraction and LSTM time-series regression	Multi-site IMUs	Moderate; Moderate; O(W × H^2^)	Pros: Significantly outperforms traditional PCA with lower RMSE (e.g., 1.963° vs. 10.353° for knee estimation); highly universal across subjects for generating adaptive device trajectories
				Cons: Requires continuous, reliable kinematic inputs from the sound limb to predict the affected limb's trajectory
Low-cost outdoor IMU system (Luo) (72)	Mahony complementary filter for attitude estimation; Gauss–Newton optimization based on kinematic hinge constraints for joint axis determination; inertial navigation system (INS) with zero-velocity update (ZUPT) for spatial parameters	Multi-site IMUs	Low; Low; O(N)	Pros: Extremely cost-effective; stable hardware synchronization via CAN bus; specifically designed for unconstrained outdoor use
				Cons: Prone to soft-tissue artifacts and sensor sliding; dynamic accuracy degrades during high-speed movement

Computational complexity configurations (Time; Memory; Big-O) were derived via author inference based on the fundamental algorithmic architectures described in each study.

*N*, total number of sampled data points in the input sequence. *τ*, number of analysis scales used in multiscale entropy calculations. *L*, length of the input window fed into neural networks. *H*, number of hidden units or feature channels in a deep learning model layer. *M*, length of the template sequence used during DTW matching algorithms. W, number of sliding windows (temporal segments) processed across the entire sequence. This depends on window size, step size, and total sequence length. C, number of input channels or feature dimensions.

### 3.6 Methods utilizing lower-limb-mounted IMUs

#### 3.6.1 Algorithm for detecting the optimal kinematic parameter signals of the legs

While IMU-based gait analysis technology has advanced and research on gait similarity has evolved, there remains a lack of consensus and standardized criteria regarding which kinematic signals maintain optimal reliability across varying conditions. This gap hinders the development of high-performance gait monitoring systems (10).

To identify robust kinematic signals, Kahlon et al. recorded shank and thigh movements (3D angular velocity and acceleration) in 30 adolescents (8–18 years) during treadmill and overground walking at self-selected, fast, and slow speeds. Gait similarity was quantified using cosine similarity, Euclidean distance, and Poincaré analysis. Medio-lateral shank angular velocity (ML shank AV) and superior–inferior shank acceleration (SI shank Acc) consistently showed high similarity in the high-frequency band, with no significant differences between treadmill and overground conditions across speeds. Poincaré analysis confirmed their robustness, whereas spatio-temporal parameters varied considerably. Thus, when walking conditions and speeds changed, ML shank AV and SI shank Acc emerged as the most reliable kinematic signals.

#### 3.6.2 CSAV algorithm

Medio-lateral shank angular velocity serves as a key observational metric in gait analysis. Mikko et al. developed and validated a novel event detection method based on the CSAV derived from IMU data. This method was evaluated using a dataset comprising nearly 25,000 gait cycles, collected from healthy adults walking under various speed and footwear conditions. Its accuracy was assessed against force plate and motion capture data and compared with existing detection methods. Results demonstrated that the CSAV method achieved high accuracy in detecting toe-off (TO), feet adjacent (FA), and tibia vertical (TBV) events and moderate accuracy in detecting heel rise (HR). A comparative analysis revealed its superior performance compared to existing methods for TO detection. The stability of the method under various speed and footwear conditions underscores its robustness (39).

#### 3.6.3 Machine learning-based leg-IMU abnormal gait classification algorithm (ML-LIGCA)

The field of gait analysis is increasingly incorporating artificial intelligence technologies, particularly machine learning methods (40–42). These quantitative tools can identify subtle gait abnormalities that are difficult to detect with traditional or subjective examinations, thereby providing greater sensitivity and objectivity for gait assessment (43).

Hwang et al. (11) developed a machine learning-based algorithm for classifying abnormal gait using leg IMUs. In this method, IMUs were securely attached to both thighs and shanks to collect data while participants walked normally, simulated a knee-injury gait, and simulated an ankle-injury gait. During data pre-processing, key gait events, such as heel strike and toe-off, were detected to segment gait cycles. Feature selection was performed using recursive feature elimination with cross-validation, and gait classification was conducted using classifiers such as support vector machine (SVM), random forest, and XGBoost.

Experimental validation demonstrated the excellent performance of the algorithm across different classification tasks. For distinguishing between normal and abnormal gait, the random forest classifier achieved 99% accuracy, precision, recall, and F1-score. In classifying knee vs. ankle-injury gaits, it attained an accuracy of 91%. These results confirm the high efficiency and accuracy of the algorithm in abnormal gait classification.

#### 3.6.4 Long short-term memory (LSTM)-based algorithm for gait phase estimation using leg-mounted IMUs (LSTM-LIGPE)

Gait phase detection in IMU-based analysis remains challenging due to interindividual variability in walking patterns and the presence of physical impairments (44). In recent years, LSTM networks have demonstrated significant progress across multiple domains (46, 47). Integrating intelligent optimization algorithms into LSTM is considered an effective strategy for enhancing model performance (48).

Sarshar et al. proposed an LSTM-based gait-phase estimation method (LSTM-LIGPE) using leg-mounted IMUs to handle complex walking patterns, noise, and individual variability. Acceleration and angular velocity data were collected from participants performing varied walking tasks. After filtering and outlier removal, features such as angular velocity magnitude were extracted; toe-off and heel-strike events were identified from the gyroscope *Z*-axis signal to annotate gait phases. A regression model was built with Keras using LSTM layers and a dense output layer (49), incorporating batch normalization and dropout. The trained algorithm demonstrated high accuracy on both training and validation sets.

#### 3.6.5 Real-time lower-limb kinematic estimation via multiple linear regression (MLR) and a Kalman filter

To overcome the inherent limitation of missing upper-leg kinematics when using only shank-mounted sensors, Wang (8) developed a novel real-time estimation framework. Rather than increasing the number of sensors, this method combines a MLR model with a lower-limb geometric model using a Kalman filter. This architecture successfully estimates the kinematics of the thigh, knee, and hip in the sagittal plane in real time using only two shank IMUs. Furthermore, the extracted physically interpretable parameters demonstrated high clinical utility, achieving 92.1% accuracy in classifying healthy adults and three distinct pathological gaits (Parkinson's disease, stroke, and peripheral neuropathy) using an SVM classifier. This approach successfully bridges the gap between sensor minimalism and the richness of multi-joint kinematic data required for clinical evaluation.

### 3.7 Methods utilizing multi-site inertial measurement unit configurations

#### 3.7.1 Unrestricted step length detection method for staircase climbing based on IMUs

While level-ground walking constitutes the primary focus of traditional gait analysis, the optimization of its assessment methods has been a consistent research focus (50, 51). Detecting key gait events during complex motor tasks, such as stair climbing, remains a significant challenge due to fundamentally altered foot kinematics. To address the limitations of existing algorithms in these scenarios, Siebers et al. (51) developed an adaptive multi-site IMU-based algorithm tailored for unrestricted step detection during both stair ascent and descent.

Rather than relying on rigid, pre-defined thresholds, the proposed method uses a multi-stage heuristic that fuses shank and foot kinematics. First, it robustly identifies the swing phase (SzerowP) by detecting distinct peaks and zero-crossings in the sagittal-plane shank angular velocity. These dynamically identified SwP boundaries are then utilized to define precise search windows. Within these temporally constrained windows, the algorithm pinpoints the exact timings of initial contact (IC) and terminal contact (TC) by extracting critical minima from multi-axis foot acceleration signals.

Crucially, the detection parameters of the algorithm are adaptively calibrated to accommodate the unique kinematic characteristics of stair navigation. By effectively filtering out invalid strides and focusing on dynamically bounded search windows, this approach demonstrates high adaptability, providing reliable technical support for real-world motion analysis and complex rehabilitation assessments (51).

#### 3.7.2 Multi-site IMU gait assessment in children with cerebral palsy across different settings

Carcreff et al. investigated this by using a multi-site IMU configuration (five sensors placed on the chest, thighs, and shanks) to evaluate children with cerebral palsy (CP) and typical development (TD) (52). Data were collected during standardized laboratory assessments and over 3 consecutive days of free-living conditions.

The algorithm used PCA on angular velocities to align sensor axes with functional movement axes, independent of precise placement. Walking bouts were detected from shank pitch angular velocity, and a double pendulum model computed spatial parameters, such as stride length. Sixteen parameters across eight domains (e.g., rhythm, stability) were extracted, with multi-site data enabling trunk-acceleration-based stability and knee-angle-based amplitude assessments. Results showed that laboratory-measured gait parameters generally exceeded those measured in daily life, with lower speed and higher variability and asymmetry. In children with CP, most parameters were highly correlated between the two environments, indicating that lab assessments reflect real-world trends. These findings provide objective evidence that children with CP perform better in supervised settings, highlighting the need to combine free-living monitoring across multiple sites with clinical assessments to inform rehabilitation decisions.

#### 3.7.3 IMU gait apraxia assessment algorithm for idiopathic normal pressure hydrocephalus (iNPH)

For specific neurological conditions characterized by complex motor dysfunctions, such as iNPH, gait apraxia is often accompanied by significant postural instability. In these scenarios, generic single-site sensor configurations are usually insufficient to capture the full spectrum of motor impairments. Multi-site configurations offer a comprehensive solution by concurrently evaluating micro-level gait kinematics and macro-level postural transitions.

A prominent example is the study conducted by Ferrari et al., which utilized a specific three-sensor configuration (one on the lower trunk and two on the bilateral shoes) to assess patients with iNPH (53). The researchers extracted 21 instrumental variables during the standardized timed-up-and-go (TUG) and 18-m walking tests. In this multi-site setup, the bilateral foot sensors accurately captured spatial–temporal gait parameters, while the lower-trunk sensor was crucial for quantifying postural transitions. The study successfully demonstrated that this multi-site IMU approach could objectively and sensitively quantify improvements in gait apraxia following clinical interventions (cerebrospinal fluid tap test and ventriculoperitoneal shunt surgery), providing significantly higher granularity than traditional clinical stopwatch assessments.

#### 3.7.4 Gait variability assessment algorithm for healthy older adults

The kinematic and kinetic analysis of gait is a vital component of the physical examination. It not only provides objective data for assessing human functional capacity and supports disability determination but also establishes a foundation for interpreting pathological manifestations through an in-depth understanding of normal gait patterns (54). Building on this foundation and following its success in accurately capturing gait parameters in specific scenarios such as stair climbing, inertial measurement unit (IMU) technology has become a focal point of current research for analyzing gait variability in healthy older adults.

Within the study of gait kinetic parameters, Rantalainen et al. conducted a pivotal study utilizing IMUs (55). Their findings indicated that IMUs worn at the waist, when estimating stride time variability via multiscale sample entropy, showed good within-week reliability and demonstrated reasonable agreement with estimates derived from force plates. In contrast, stride time variability estimates based on ankle-worn IMUs, while exhibiting moderate reliability, showed poor concurrent validity when compared to force plate assessments.

To evaluate gait variability and complexity, participants wore both ankle and waist IMUs during habitual walking over force plates. The ankle-worn sensors showed limited validity in stride time variability due to inaccuracies in heel-strike detection. Conversely, waist-worn IMUs using a gradient descent algorithm demonstrated strong agreement with the force plate reference. In addition, waist IMU data effectively quantified gait complexity using entropy metrics, with refined composite multiscale entropy (RCME) proving highly sensitive to variability.

Beyond single-sensor applications, system-level validation is essential for multi-site IMU fusion systems. Comparisons with optical motion capture indicate that while multi-site systems accurately capture hip kinematics, significant discrepancies remain for knee and ankle measurements, particularly during the swing phase. Thus, while promising for clinical gait analysis, these systems require rigorous joint- and phase-specific validation prior to widespread real-world deployment (56).

#### 3.7.5 Weighted-average ensemble classification model

Beyond evaluating postural transitions, comprehensive multi-site configurations are highly effective for complex differential diagnoses. For instance, Lin et al. developed a weighted-average ensemble classification model using a 10-sensor IMU setup combined with machine learning (e.g., support vector machine) to analyze segmented timed-up-and-go (TUG) tests (57). By capturing subtle multi-dimensional kinematic differences—particularly arm-swing symmetry during straight walking—this model successfully discriminated early-stage Parkinson's disease from essential tremor with 75.8% accuracy, highlighting its critical value for nuanced clinical diagnostics.

#### 3.7.6 ConvLSTM network

Leveraging a high-density 11-sensor array across the lower and upper limbs, Kreuzer and Munz developed the ConvLSTM network for precise gait phase recognition (45). Instead of relying on traditional heuristic rules or fixed thresholds, they fed synchronized multi-channel time-series data into this combined convolutional and long short-term memory network. By extracting rich spatial and temporal features, the ConvLSTM network successfully classified five distinct gait phases with a mean accuracy of 92.29% across unseen participants. Crucially, deploying this comprehensive multi-site array enables the systematic evaluation of sensor redundancy. A feature importance analysis of convolutional activation maps revealed that thigh-mounted gyroscope data contributed most to classification. This demonstrates how combining high-density networks with deep learning can both achieve robust multi-phase segmentation and inform data-driven sensor reduction strategies for streamlined clinical applications.

#### 3.7.7 LSTM-based synergy modeling

Beyond phase segmentation, neural networks are increasingly utilized to model gait synergy for assistive device control. Liang et al. employed a LSTM network to model both interlimb and intralimb synergies, mapping continuous relative phase dynamics across healthy, stroke, and amputee participants (46). By utilizing sound-limb kinematics (angular velocities and accelerations) from a seven-sensor lower-limb array as inputs, the LSTM model effectively predicted the contralateral hip and knee joint angles. In interparticipant simulations, the LSTM framework demonstrated significant superiority over traditional principal component analysis (PCA), reducing the root mean square error (RMSE) from 5.050° to 0.796° for hip interlimb estimation. This demonstrates that multi-site LSTM models can robustly generate adaptive, wearer-specific reference trajectories, offering a highly accurate data-driven strategy to improve human–machine interaction in lower-limb exoskeletons and prostheses.

#### 3.7.8 Low-cost outdoor IMU system

Compared with computationally intensive deep learning models, classical model-driven algorithms remain highly relevant, particularly for low-cost, outdoor applications where embedded processing constraints are strict. Luo demonstrated a comprehensive multi-site algorithmic pipeline, referred to as the low-cost outdoor IMU system (72), utilizing a seven-node IMU network. Rather than relying on data-driven black boxes, this approach employs a Mahony complementary filter for computationally efficient attitude estimation. To calculate joint angles without precise sensor-to-bone alignment, the algorithm uses a Gauss–Newton optimization method based on kinematic hinge constraints to determine the joint rotation axis. Furthermore, to extract spatial parameters such as stride length, the system integrates an inertial navigation system with a zero-velocity update (ZUPT) algorithm, resetting the integration drift during the threshold-detected stance phase. Although this classical sensor fusion framework is susceptible to soft-tissue artifacts during high-speed movement (yielding a dynamic knee-angle average error of 6.4°), it demonstrates that low-cost embedded systems can achieve reliable outdoor gait tracking without relying on costly commercial algorithms or external optical references.

### 3.8 Methods utilizing foot-mounted inertial measurement units

#### 3.8.1 Orientation-invariant spatio-temporal gait analysis algorithm using foot-worn IMUs (OI-STGA)

Conventional foot IMU gait algorithms suffer from accuracy loss due to arbitrary sensor rotation, especially during complex locomotion. To resolve this limitation, Guimarães et al. proposed the OI-STGA pipeline free of strict sensor alignment constraints (1). The workflow centers on optimized zero-velocity interval detection via angular rate energy detection, followed by orientation estimation comparing Madgwick and Euston complementary filters. After gravity compensation, dual integration strategies (linear drift elimination and reverse integration) are applied to compute spatial displacement, with leveling correction to mitigate cumulative bias. Gait events, including initial contact (FC) and foot off (FO), are extracted from acceleration signals, then used to derive full spatio-temporal gait parameters. The dataset was split for model tuning and independent validation. Comparative tests identified the Euston filter paired with active leveling correction as the optimal combination. Simulated sensor rotation experiments verified strong orientation robustness, and the algorithm achieved consistent agreement with reference measurements across standard gait metrics. This method delivers stable, easy-to-deploy gait quantification suitable for both clinical assessments and unsupervised daily monitoring.

#### 3.8.2 SDI-step algorithm

Building on this foundation, Muthukrishnanet al. also focused on the precise acquisition of spatio-temporal gait parameters. In contrast to the aforementioned study, Muthukrishnan et al. developed the SDI-step algorithm, which prioritizes the precise calculation of step length and step time by employing a distinctive data-processing and computational workflow. Measurements obtained from wearable IMU signals processed by the SDI-step algorithm demonstrated strong agreement with those from widely used laboratory-based systems, establishing it as a valid tool for measuring spatio-temporal gait parameters. The SDI-step algorithm is executed through nine sequential steps.

A comparative analysis of the outcomes reveals that while both approaches employ IMUs for gait analysis, their emphases differ. The OI-STGA algorithm (Guimarães et al.) primarily addresses the need for orientation invariance and stability across multiple gait parameters. Conversely, the SDI-step algorithm excels in the accuracy of step length and step time calculation, demonstrates good applicability across diverse populations, and assists clinicians in better understanding diseases and formulating more effective rehabilitation plans (58).

#### 3.8.3 Self-adaptive algorithm for detecting key contact points during running, based on foot-worn IMUs

The increasing popularity of running and the rapid advancement of micro-electro-mechanical systems (MEMS) have enabled portable wireless sensors to facilitate on-site monitoring and analysis of running gait parameters (59). Yang et al. proposed a self-adaptive algorithm for detecting key contact points during running based on foot-worn IMUs.

Regarding data processing, a pre-processing algorithm was first designed to detect the running cycle, determining its start and end times to ensure accurate subsequent detection of initial contact (IC) and terminal contact (TC). In terms of algorithm design, the accelerometer-based method utilized *Z*-axis acceleration data. The square of this signal was calculated, and the peak value of the resultant foot acceleration was identified as the IC detection point. Within the 25%–75% region of the stride, the TC event was determined based on the acceleration trend and a predefined threshold (2*g*). The gyroscope-based method initially detected the mid-swing (MS) phase. A low-pass filter was applied to enhance the MS peak, after which specific rules were followed to determine the positions of TC, MS, and IC.

Through this unique design, the adaptive algorithm demonstrated excellent performance during running. Its primary limitation is that its performance is influenced by running speed and depends on the foot strike pattern.

#### 3.8.4 Martin and Salaun algorithm

Gait analysis is crucial for assessing individual motor function, and accurate measurement of parameters such as ankle dorsiflexion is particularly important. Individuals with limited ankle dorsiflexion often exhibit abnormal lower-limb movement patterns during walking (60). To address this, Feuvrier et al. employed the Martin and Salaun algorithm to process inertial measurement unit (IMU) data. This algorithm first processes the raw data, which includes angular velocity, acceleration, and magnetic field information. It calculates parameters such as rotation angles and linear velocity through integration while applying specific filters to remove noise and ensure the integrity of subsequent analysis. Subsequently, the sensor data are transformed into a coordinate system suitable for human motion analysis. The processed data can then be used to analyze gait parameters, including ankle dorsiflexion angle at heel-strike and mid-swing, stride length, walking speed, and maximum ankle dorsiflexion velocity. The algorithm achieves particularly high accuracy in measuring ankle dorsiflexion. Its advantages include enhanced reliability, low computational demands, and consideration of the characteristics of all integrated sensors (61).

#### 3.8.5 gait event detection algorithm for robot-assisted gait training in stroke patients

Stroke remains the second leading cause of death globally and the third leading combined factor for mortality and disability, as measured by disability-adjusted life years (DALYs) (62). Studies indicate that approximately 90% of stroke survivors experience functional impairment, with motor dysfunction being the most prevalent. This significantly compromises quality of life of patients and increases societal healthcare burdens (63). To ameliorate this situation, Schicketmueller et al. developed a foot-mounted inertial measurement unit (IMU)-based gait event detection algorithm. This innovation accurately identifies a patient's gait cycles in real-time by parsing kinematic data during robot-assisted gait training. It aims to expand the use of robot-assisted rehabilitation in areas such as functional electrical stimulation (FES), thereby offering more effective rehabilitation interventions for post-stroke motor dysfunction (64).

Using bilateral foot-mounted IMUs, the algorithm employs a finite state machine to sequentially detect four key gait events based on acceleration and angular velocity: initial contact (IC), full contact (FC), heel-off (HO), and toe-off (TO). The detection logic relies on specific kinematic triggers; for example, IC is captured when the angular velocity transitions from a negative range to a sustained positive value with a positive slope. The remaining events are subsequently identified following the logic of the state machine'.

To ensure robustness, the algorithm integrates a stringent error-handling mechanism. Gait events must occur in strict sequential order; any anomaly immediately resets the detection process. Furthermore, temporal constraints—such as a minimum required interval between IC and HO—are enforced to suppress false positives. This rigorous framework ensures the extraction of highly reliable kinematic data to support personalized rehabilitation planning.

#### 3.8.6 Parkinson's disease standardized gait test detection algorithm based on foot IMUs

Parkinson's disease (PD) is a chronic and progressive neurological disorder (65). The analysis of gait data is as a crucial tool for clinicians to diagnose and manage PD and similar conditions (66). Given that gait impairments are prevalent among PD patients and manifest differently across disease stages, gait analysis holds significant clinical value for auxiliary diagnosis (67).

Beyond standardized gait-test detection in PD (38, 68), foot-/insole-based wearable sensor studies also support clinical gait characterization in Parkinsonian disorders. A comparative study including PD, Parkinson-variant multiple system atrophy (MSA-P), and older healthy controls quantified gait variability and spatio-temporal parameters under different walking speeds and reported higher gait variability in MSA-P than in PD in several measures, indicating the importance of foot-/insole-based sensing for differentiating gait instability across Parkinsonian syndromes (69).

More recent studies have further extended foot-IMU gait event detection to free-living, unsupervised walking conditions. For example, a Mobilise-D Consortium study used bilateral foot IMUs with pressure insoles as reference and showed high performance in detecting gait events, with good agreement in derived temporal gait parameters (70). This finding supports the ecological validity of foot-IMU-based gait analysis and highlights its potential for real-world mobility monitoring.

Overall, foot-IMU-based approaches in Parkinsonian gait assessment have evolved from standardized gait sequence/event detection to broader clinical gait assessment and real-world gait monitoring. However, further validation is still needed to improve generalizability across cohorts and testing conditions.

#### 3.8.7 Foot-IMU-based walking bout detection in real-life settings

Laboratory-based or standardized walking tests may not fully reflect gait performance in daily life. Therefore, real-life walking bout detection is an important step toward improving the ecological validity of foot-IMU-based gait assessment. A recent study proposed and validated a robust walking-bout detection approach using a single foot-worn IMU, with performance evaluated against the INDIP reference system in real-life conditions (71). The study showed that foot-IMU-based methods can achieve reliable walking bout detection in unsupervised settings and can support the subsequent extraction of real-world gait outcomes. These findings highlight the potential of foot-mounted IMUs for long-term ambulatory monitoring, although further validation is still needed in larger cohorts and in patients with more diverse pathological gait patterns.

### 3.9 Methods utilizing chest-mounted inertial measurement units

#### 3.9.1 KF+ model algorithm

The KF+ model algorithm uses stride length as a key metric. Although long-term monitoring using IMUs is cost-effective, accurately obtaining spatial gait parameters remains challenging. This study focused on slow walking speeds (1–4 km/h) and specifically investigated a model-based algorithm utilizing a chest-worn, pendant-style IMU.

To estimate stride length, the methodology integrated a MEMS-IMU and a barometric pressure sensor (BPS). Filtered 3D acceleration and BPS-derived altitude data were first fused to precisely determine vertical position. Subsequently, a Kalman filter (KF) was used to estimate the vertical displacement. By leveraging specific gait events to correct integration drift, this KF approach achieved significantly higher accuracy than traditional double-integration methods. Stride length was then computed via a modified inverted pendulum model, identifying support phases through vertical acceleration zero-crossings. Although robust, prediction errors increased at walking speeds below 2 km/h. This performance degradation was primarily attributed to vertical displacement overestimation and the inherent kinematic limitations of the simplified model at low velocities.

Overall, this study provides an effective method for gait analysis using a chest-worn pendant IMU, particularly demonstrating good performance within the 2–4 km/h speed range. This algorithmic approach offers a compelling approach to unobtrusively monitor stride length—and consequently, gait stability—in daily life (73).

### 3.10 Methods utilizing lower-back-worn inertial measurement units

#### 3.10.1 Mobilise-D chronic obstructive pulmonary disease (COPD) gait analysis algorithm using a waist-worn IMU

Delgado-Ortiz et al. (74) assessed real-world gait in 549 COPD patients and 19 healthy older adults using a waist-mounted IMU (lower back) with 7 days of continuous wear. Data were processed via the standardized Mobilise-D pipeline to derive 15 digital mobility outcomes (DMOs) encompassing gait pace, rhythm, and bout-to-bout variability.

All DMOs were normally distributed. With increasing COPD severity (GOLD grades, ABE groups, mMRC scores), walking speed, cadence, and stride length declined significantly (*p* for trend < 0.001). After adjustment, these associations remained significant, while bout-to-bout variability was only associated with mMRC grades. Compared to healthy peers, COPD patients walked slower during long walking bouts (>30 s) (0.83 ± 0.12 vs. 0.90 ± 0.12 m·s^−1^, *p* = 0.041), had lower maximum speed (0.99 ± 0.18 vs. 1.12 ± 0.18 m·s^−1^, *p* = 0.015), and reduced cadence (91 ± 7 vs. 100 ± 9 steps·min^−1^, *p* = 0.039). No differences were observed for short bouts or stride parameters. These findings demonstrate that waist-worn IMUs capture clinically meaningful gait impairments in COPD under free-living conditions, with deterioration paralleling disease progression.

#### 3.10.2 Lower-back assessment for fear of falling in Parkinson's disease

For single-sensor configurations on the lower trunk, evaluating the discrepancy between clinical capacity and daily-life performance is crucial for patients with complex neurological symptoms. Atrsaei et al. proposed a comprehensive lower-back IMU mobility assessment algorithm to investigate the effect of fear of falling (FOF) on patients with Parkinson's disease (75). Utilizing a single IMU positioned on the lower back, the researchers successfully extracted multi-domain mobility parameters—including gait speed, sit-to-stand transitions, and turning kinematics—across both supervised clinical tests (such as the timed-up-and-go and 5-times sit-to-stand) and 14 days of unsupervised home monitoring. The study demonstrated that while lab-based parameters (particularly turning peak angular velocity) showed high discriminative power, combining these capacity metrics with daily-activity performance data significantly improved the classification accuracy for identifying FOF (achieving an AUC of 0.75). Interestingly, continuous lower-back monitoring revealed a behavioral discrepancy: PD patients with FOF exhibited more cautious movements in the laboratory but adopted relatively incautious fast-walking patterns in their familiar home environments. This finding highlights the unique advantage of lower-back IMUs in capturing the hidden gap between what patients can do in a clinic and what they actually do at home, providing a holistic view of fall risks that single-setting assessments might easily miss.

#### 3.10.3 Lower-back real-world gait assessment in multi-pathological populations

Further extending the application of lower-back IMUs to diverse pathological populations, Micó-Amigo et al. conducted an extensive validation study within the Mobilise-D Consortium (76). The researchers evaluated multiple algorithms for extracting digital mobility outcomes (DMOs)—such as gait sequence detection, initial contact, cadence, and stride length—using a single lower-back sensor during 2.5 h of unconstrained real-world monitoring. The study included 108 participants across six distinct cohorts, including Parkinson's disease (PD), multiple sclerosis (MS), COPD, congestive heart failure (CHF), proximal femoral fracture (PFF), and healthy adults. By comparing single-sensor data with a gold-standard multi-sensor reference system (INDIP) equipped with pressure insoles, they demonstrated that lower-back IMUs provide robust DMO estimates in free-living environments. Crucially, the findings revealed that algorithm performance is highly cohort-specific; accuracy degrades significantly for slow walkers and during short walking bouts. This finding underscores the need to tailor lower-back algorithms to specific disease profiles to ensure clinical reliability in real-world gait assessment.

#### 3.10.4 Waist-worn slow-gait estimation algorithm for sarcopenia

Monitoring real-world gait in frail, elderly populations presents unique challenges, primarily because algorithms trained on healthy adults consistently overestimate walking speed in slow-walking pathological cohorts. To address this gap, Mueller et al. developed and validated a waist-worn IMU algorithm specifically tailored for slow and frail walkers, focusing on older adults with sarcopenia (77). The algorithm uses a short-time Fourier transform to extract dominant frequencies and applies a Hilbert transform to determine frequency, phase, and amplitude across all three axes, then projects the gait speed using a linear model. The researchers conducted an independent validation study using a realistic clinical “parcours” course (*n* = 26) and a large-scale, multi-site interventional clinical trial involving 217 sarcopenic patients monitored continuously for 25 weeks. The results demonstrated that this multi-axis approach accurately captures real-world gait speeds—even below 0.5 m/s—with a residual standard error of only 0.08 m/s compared with clinical reference tests (e.g., 4 mWT, 6 MWT, 400 mWT). This large-scale validation confirms that waist-worn IMUs, when paired with appropriately tuned signal-processing pipelines, can reliably quantify mobility decline and slow-gait parameters in frail populations during free-living conditions.

#### 3.10.5 Mobilise-D lower-back computational pipeline for hip fracture recovery

To further demonstrate the large-scale clinical utility of lower-back IMUs in specific post-operative recovery scenarios, Becker et al. applied the validated Mobilise-D lower-back computational pipeline to monitor real-world walking in elderly patients recovering from proximal femoral fractures (PFFs), commonly known as hip fractures (78). Utilizing a single lower-back wearable device (e.g., Axivity AX6 or McRoberts MoveMonitor), the researchers continuously monitored a massive cohort of 564 PFF participants (mean age 77.5 years) for 7 days across four distinct longitudinal recovery phases: acute, post-acute, extended recovery, and long-term recovery. The algorithm effectively identified walking bouts and extracted comprehensive digital mobility outcomes (DMOs), particularly focusing on walking amount, pace (e.g., walking speed, cadence), and pattern (e.g., bout distribution). The study revealed significant and measurable variations in these DMOs across the different stages of healing, highlighting that the lower-back computational pipeline can provide highly granular, real-world insights into the mobility recovery trajectories of elderly hip fracture survivors. This large-scale, multi-phase application underscores the capability of the algorithm to serve as a robust prognostic tool for tracking rehabilitation progress in extremely frail surgical cohorts.

#### 3.10.6 Mobilise-D computational pipeline for healthy aging mobility benchmarks

While pathological cohorts highlight the clinical utility of lower-back IMUs, establishing normative benchmarks for healthy aging is equally critical for interpreting real-world digital mobility outcomes (DMOs). Albites-Sanabria et al. addressed this need by applying the validated Mobilise-D computational pipeline for healthy aging mobility benchmarks within the InCHIANTI study (79). Using a single lower-back sensor over a 7-day free-living monitoring period, the researchers analyzed data from 200 community-dwelling older adults (aged 65–94 years) to extract 24 distinct DMOs that cover walking activity, pace, rhythm, and bout-to-bout variability. The study provided comprehensive reference values and revealed complex, non-linear trajectories of mobility decline associated with aging. Notably, generalized additive models (GAMs) showed that gait parameters, such as real-world walking speed and stride length, remain relatively stable until a critical age threshold, after which they exhibit an accelerated, stepwise decline, with distinct gender differences. By providing robust normative data for real-world walking, this study bridges a vital gap, enabling clinicians to accurately distinguish between natural age-related mobility changes and pathological decline using lower-back wearable sensors.

### 3.11 Methods utilizing wrist-mounted inertial measurement units

#### 3.11.1 CNN-based wrist gait detection algorithm

Wrist-worn sensors offer superior user compliance for continuous, long-term monitoring, making them highly desirable for real-world gait analysis. However, extracting reliable gait sequences from the wrist is technically challenging due to the confounding noise of unstructured daily arm movements. Brand et al. (80) addressed this challenge by evaluating various machine learning frameworks, including a novel unsupervised anomaly detection algorithm and a supervised deep convolutional neural network (DCNN), for wrist-based gait detection (80). Using data from 30 older adults (12 community-dwelling older adults and 18 individuals with Parkinson's disease) monitored over a 10-day free-living period, the researchers rigorously validated the algorithm outputs. The study highlighted the inherent tradeoffs between precision and recall in highly imbalanced real-world datasets, with the DCNN algorithm ultimately delivering the highest performance (AUC of 0.94 for healthy older adults and 0.89 for PD patients). Crucially, by optimizing classification thresholds to prioritize high precision, the wrist-based DCNN model achieved strong correlations (*r* > 0.8) with the reference standard for estimating daily walking time and successfully differentiated gait quality metrics between cohorts. By validating these advanced computational methods, this study demonstrates the feasibility of quantifying continuous, real-world gait behavior using highly accepted wrist-worn devices, overcoming the traditional limitations of arm-movement noise.

The robustness of this DCNN model was further corroborated by an independent real-world validation by Kluge et al. (81). Evaluating 10 wrist-based algorithms across 83 patients from 5 pathological cohorts, the Brand (80) approach achieved the best overall performance. It demonstrated excellent specificity (>0.95) in rejecting non-gait arm movements during unconstrained activities. Although detection sensitivity decreased among frail patients using bilateral walking aids, these findings collectively confirm that wrist-worn devices can reliably quantify continuous real-world gait by effectively mitigating traditional arm-movement noise.

#### 3.11.2 Self-supervised deep learning for wrist-worn sensors

While multi-site IMU configurations provide comprehensive biomechanical data, their complexity often limits long-term patient compliance in free-living environments. To address this, recent advancements in deep learning have enabled the extraction of precise spatial-temporal gait metrics from a single wrist-worn accelerometer. Brand et al. introduced ElderNet, a self-supervised learning (SSL) framework based on a ResNet architecture (82). By leveraging large unlabeled datasets (e.g., UK Biobank) for pre-training, ElderNet learns robust representations of human movement before fine-tuning on specific clinical cohorts. Validated against a multi-sensor reference system integrating pressure insoles, ElderNet accurately estimated gait speed (mean absolute error of 8.8 cm/s), cadence, and stride length in older adults and patients with diverse impairments, including Parkinson's disease and multiple sclerosis. Furthermore, applying ElderNet to multi-day, free-living recordings demonstrated that wrist-derived gait metrics could classify mobility disability with high accuracy (AUC = 0.80), significantly outperforming conventional supervised clinical assessments. This data-driven approach highlights the immense potential of combining highly compliant wearable locations with advanced SSL algorithms to achieve scalable, continuous monitoring in real-world applications.

### 3.12 Effectiveness of IMU-based gait screening methods

In IMU-based gait analysis, various methodologies have been employed across studies to assess gait and identify valid signals. Direct comparisons between these approaches are often challenging due to inconsistencies in experimental conditions, as summarized in Table 3. This review synthesizes these findings to provide a structured framework and practical recommendations for selecting appropriate IMU-based gait analysis solutions.

**Table 3:** Methodological profiles and clinical evidence matrices of IMU-based gait assessment algorithms.

Algorithm	Sensor placement	Study design	Sample size	Population	Walking task	Setting	Reference standard	Main validation metric	Key limitations
Martin and Salaun algorithm (61)	Foot	Validation study	*n* = 26	Adults (45–84 years, 31% women)	10-m walk with two half-turns	Laboratory	Vicon OMC system	RMSE < 6.1°, MAE < 4.3° (dispersion 95% CI ± 12°)	Performance during non-straight-line trajectories remains unstable
Unrestricted step length detection method for staircase climbing based on IMU (51)	Multiple sites	Validation study	*n* = 35	Healthy adults (23–29 years, 46% women)	Walking, stair ascent, and descent	Laboratory (controlled staircase)	Noraxon and Figueiredo commercial algorithms	100% success in detecting all six strides during stair climbing	Typical errors included premature detection of IC or TC before contralateral IC
Algorithm for detecting the optimal kinematic parameter signals of the legs (10)	Lower limb	Cross-sectional	*n* = 30	Adolescents (8–18 years, 33% women)	3-min walking (self-selected, fast, and slow)	Treadmill/overground	Distance-based similarity measures	ML shank AV and SI shank Acc identified as most robust signals	Validated only in healthy/typical adolescents; lacks validation in pathological populations
KF+ model algorithm (73)	Chest	Validation study	*n* = 10	Adults (29.8 ± 4.24 years, 33% women)	Treadmill walking (≥2 km/h)	Laboratory	Zebris FDM-T treadmill	Relative error 7.95% at speeds ≥ 2 km/h	Requires manual input of leg length; significant error increases at very low speeds
SDI-step algorithm (2)	Foot	Validation study	*n* = 14	Young adults and PD patients (23.6–30.5 years, 40–56% women)	Overground straight-line walking	Laboratory	GAITRite electronic walkway/Photoelectric system	Moderate-to-excellent agreement (ICC ≥ 0.81)	Generalizability of drift constraints across varying cadences requires further validation
Gait variability assessment algorithm for healthy older adults (55)	Multiple sites	Validation study	*n* = 27	Older adults (∼74 years, 63% women)	Habitual speed walking (3 mins)	Laboratory	Custom force plate array	Waist IMU ICC = 0.66–0.82; RCME significantly correlated at coarse scales	Ankle-worn IMU showed lower reliability and validity for stride time variability
Cumulative shank angular velocity (CSAV) algorithm (39)	Lower limb	Validation study	*n* = 15	Adults (23.7 ± 3.5 years, 67% women)	Varying speeds and footwear	Laboratory	Vicon OMC system and force plates	Mean errors ≤ 1.6 ms (ICCs ≥ 0.988) for IC, TO, FA, TBV events	Heel rise (HR) event had a larger mean error (0.9 ± 29.3 ms) and lower ICC
Long short-term memory (LSTM)-based algorithm for gait phase estimation using leg-mounted IMUs (LSTM-LIGPE) (44)	Lower limb	Model validation	*n* = 64	Healthy, glaucoma, cLBP patients (68.9–70.5 years)	Various walking tasks	Laboratory	Foot switches (gold standard)	Accuracy 0.9981 (train), 0.9977 (val), 0.9945 (test)	Slightly lower accuracy observed in cLBP cohort; high memory/data footprint
Orientation-invariant spatio-temporal gait analysis algorithm using foot-worn IMUs (OI-STGA) (1)	Foot	Validation study	*n* = 26	Adults (29.2 ± 5.3 years, 50% women)	Overground walking with turns	Laboratory	Vicon OMC system	Orientation invariance confirmed (RMSD = 0.0); MTC relative error 0.2 ± 0.8 cm	Larger errors for swing width and MTC; complex coordinate transformation pipeline
Machine learning-based leg-IMU abnormal gait classification algorithm (ML-LIGCA) (11)	Lower limb	Classification study	*n* = 10	Males (39.4 ± 10.3 years, 0% women)	Normal vs. simulated injury gaits	Laboratory	Walkway system	RF model accuracy 99% (binary), 91% (three-class)	Validated only on simulated injury gaits, lacking real pathological cases
Real-time lower-limb kinematic estimation via MLR and Kalman flter (Wang) (8)	Lower limb	Validation and classification	*n* = 56	Four cohorts (PD, stroke, PN, healthy; 34–71 years, 39% women)	Overground straight-line walking (≥12 m)	Laboratory	Vicon OMC system	Stride length MAE ∼0.02 m; 4-class gait classification accuracy 92.1%	Small sample sizes in specific pathological subgroups; validation restricted to sagittal-plane straight-line walking
Optimization of IMU sensor placement for the measurement of lower limb joint kinematics (Niswander et al.) (9)	Multiple sites	Validation study	*n* = 7	Healthy adults (26.0 ± 4.0 yrs, 43% women)	Timed-up-and-go (TUG) test	Laboratory	Optical motion capture system	Bias and RMSE varied by sensor position and movement type	Small sample size (*n* = 7); limited to healthy participants
Self-adaptive algorithm for detecting key contact points during running, based on foot-worn IMUs (59)	Foot	Validation study	*n* = 38	Runners	Running at varying speeds	Laboratory	Video recording	100% agreement at 0.05 s tolerance; error range 0.021–0.036 s	Consistency lower for forefoot strike patterns; influenced by running speed
Gait event detection algorithm for robot-assisted gait training in stroke patients (64)	Foot	Validation study	*n* = 10	Stroke patients	Robot-assisted gait training	Clinical	Manual count	Detection rate > 80% (Lyra), >90% (Lokomat)	Narrow kinematic scope; requires optimization for complex non-linear movements.
Parkinson's disease standardized gait test detection algorithm based on foot IMU (68)	Foot	Validation study	*n* = 12	PD patients (64.9 ± 9.3 years, 17% women)	3× consecutive 4 × 10MWT	Free-living	Manual annotation/Video/App tags	F1-score = 94.0% for test decomposition; Parameter correlations 0.94–0.987	Requires fixed template definitions; susceptible to varying walking speeds
Foot-IMU-based walking bout detection in real-life settings (71)	Foot	Validation study	*n* = 18	Healthy young and older adults	2.5 h free-living activities	Free-living	INDIP multi-sensor reference system	Accuracy >90% and sensitivity >98% for walking detection	Performance in severe pathological gait patterns requires further validation
Foot-IMU-based deep learning gait event detection in mobility-limiting diseases (70)	Foot	Validation study	*n* = 99	Six cohorts (CHF, COPD, MS, PD, etc.; 42–83 years)	Habitual/free-living walking	Free-living	Pressure insoles and foot IMUs	Recall 98%/precision 96% for ICs; derived parameters show good agreement	“Black-box” architecture lacks direct biomechanical interpretability
Mobilise-D COPD gait analysis algorithm using a waist-worn IMU (74)	Waist (lower back)	Cross-sectional	*n* = 568	COPD patients and healthy adults (67.6–71 years, 36.8%–47% women)	7-day continuous monitoring	Free-living	Standardised Mobilise-D pipeline	Walking speed, cadence, and stride length worsened with COPD severity (*p* < 0.001)	May fail to capture meaningful impairments during ultra-short walking bouts
Multi-site IMU gait assessment in children with CP (52)	Multiple sites	Observational	*n* = 28	Children with CP and TD (8–20 years, 57% women)	Lab assessment vs. 3-day monitoring	Lab vs. free-living	Standardized clinical gait analysis (CGA)	12 of 16 gait parameters showed good-to-very good correlations in CP	Double pendulum mathematical model relies on precise manual measurements
Weighted average ensemble classification model (Lin et al.) (57)	Multiple sites	Classification	*n* = 164	Early PD and ET patients (∼58 years)	Segmented TUG test	Clinical	Standard clinical diagnosis	Accuracy: 75.8%, AUC: 0.823 for PD vs. ET	Feature engineering burden is high; 10-sensor setup is cumbersome
CNN-based wrist gait detection algorithm (80)	Wrist	Validation study	*n* = 32	Older adults and PD patients (73.2 ± 6.7 years, 57% women)	Free-living activities	Free-living	Standardized lower-back IMUs	High accuracy identifying walking bouts, outperforming traditional algorithms	High computational resource demands; requires extensive dataset diversity
Self-supervised deep learning for wrist-worn sensors (Brand et al., 2025) (82)	Wrist	Validation and clinical utility study	*n* = 85 (validation); *n* = 819 (clinical utility)	Older adults, patients with gait impairments (PD, MS, COPD, CHF, PFF), and young adults	Free-living activities (monitoring from 2.5 h up to 10 days)	Free-living	INDIP multi-sensor system	Gait speed MAE of 8.82 cm/s and ICC of 0.87; Classified mobility disability with an AUC of 0.80	Wrist movements inherently contain high non-gait noise; model was specifically optimized for older adults, showing reduced performance in younger/healthier cohorts (e.g., the MS cohort) without specific retraining
				Mobilise-D: ∼46.5–79.6 years, 41% women					
				RUSH MAP: 83.3 ± 7.4$ years, 23% women					
				Young adults: 29.6 ± 7.8 years					
IMU gait apraxia assessment algorithm for iNPH (53)	Multiple sites	Pre/post-intervention	*n* = 42	iNPH patients (75.2 ± 4.0 years, 64% women)	TUG, 18 mW	Clinical	Standard clinical stopwatch assessments	Objectively quantified improvements; higher sensitivity than stopwatch	High signal synchronization overhead increases pre-processing complexity
Lower-back IMU mobility assessment algorithm for fear of falling in PD (75)	Lower back	Predictive	*n* = 26	PD patients (64–65 years, 81% women)	TUG, 5xSTS vs. 14-day home monitoring	Clinic vs. free-living	Standardized clinical assessments	Combined lab/daily features yielded highest discriminative power (AUC = 0.75)	Turning detection logic is prone to misclassifying general non-locomotion rotations
Multi-cohort lower-back real-world gait assessment algorithms (76)	Lower back	Validation study	*n* = 108	Six cohorts (51.5–80.3 years, 42% women)	2.5 h free-living activities	Free-living	INDIP reference system	Robust estimation of key DMOs in real-world settings	Algorithm performance degrades significantly for irregular slow walkers and short bouts
Waist-worn slow-gait estimation algorithm for sarcopenia (Mueller et al.) (77)	Waist	Validation/trial	*n* = 243	Sarcopenia/frail older adults (>60 years, 58% women)	Parcours and standard mobility tests	Clinical	Reference device/Standard tests	Strongly correlated (*r* = 0.84, residual standard error=0.08 m/s)	Linear projection model is over-fitted to low-frequency data and may fail on fast walking
Mobilise-D lower-back computational pipeline for hip fracture recovery (78)	Lower back	Longitudinal	*n* = 564	Elderly recovering from PFF (77.5 ± 9.6 years, 66% women)	7-day monitoring across four recovery phases	Free-living	Comparison across recovery phases	Captured highly granular variations in DMOs across recovery phases	Relies heavily on predefined minimum wear-time criteria, risking data exclusion
Mobilise-D healthy aging benchmark pipeline (Albites-Sanabria et al.) (79)	Lower back	Observational benchmark study	*n* = 200	Community-dwelling older adults (65–94 years, 54% women)	7-day continuous free-living monitoring	Real-world/daily-living	N/A (employed the previously validated Mobilise-D algorithmic pipeline)	Established normative real-world gait benchmarks; quantified non-linear age and sex-related declines in gait parameters	Cross-sectional design limits causal longitudinal inferences; excluded individuals heavily reliant on walking aids, potentially introducing selection bias
Single- and dual-task gait performance and their diagnostic value in early-stage Parkinson's disease (3)	Lower limb/foot	Diagnostic study	*n* = 138	Early-stage PD (66.5 ± 9.2 years, 43% women) and HC	Single-task (ST) and dual-task (DT) 7-m walk	Clinical	Standard clinical diagnosis	Combined ST and DT parameters achieved AUC = 0.924 (sensitivity 85.4%, specificity 92.7%)	Cross-sectional design lacks longitudinal tracking; relies on only one specific cognitive dual-task
ConvLSTM network (45)	Multiple sites	Validation study (LOOCV)	*n* = 11	Healthy adults (20–34 years)	Treadmill walking	Laboratory	Instrumented pressure plate treadmill (Zebris FDM-T)	Mean accuracy of 92.29% for 5-phase classification on unseenparticipants; >99% accuracy if a 24 ms transition tolerance is accepted	Evaluated solely on a small cohort of young, healthy participants without pathological gait considerations; testing restricted to treadmill walking
LSTM-based synergy modeling (46)	Multiple sites	Validation study (LOOCV)	*n* = 27	Healthy adults (*n* = 8, 0% women); stroke patients (*n* = 11, gender unreported); unilateral transfemoral amputees (*n* = 8, gender unreported)	Level ground walking at self-selected speeds	Laboratory and clinical	Original measured joint angles from IMU	Interlimb RMSE: 0.796° (hip), 1.963° (knee); intralimb RMSE: 3.894° (knee)	Validation relies on offline simulation; real-time physical testing on active assistive devices is pending
Low-cost outdoor IMU system (Luo) (72)	Multi-site	Validation study	*n* = 5	Healthy adults (3 men, 2 women)	Overground walking at low, mid, and high speeds	Laboratory and outdoor	VICON optoelectronic system and footprint measurement method	Dynamic knee angle average error 6.4°; stride length error < 10 cm; stance phase detection max delay 53 ms	Evaluated solely on a very small healthy cohort (*n* = 5); dynamic kinematic accuracy degrades notably due to soft-tissue artifacts during movement

#### 3.12.1 Effectiveness of key kinematic signals and gait event detection methods

Foundational studies have established robust frameworks for gait event detection (Table 1). For instance, Catalfamo et al. (24) demonstrated that a single shank-mounted gyroscope employing peak-detection algorithms reliably extracts gait phases across complex terrains, while Jasiewicz et al. (28) validated the accuracy of linear accelerometer and angular velocity thresholds for event timing. However, as monitoring shifts toward unconstrained real-world environments, identifying kinematic signals that maintain stability across highly variable conditions remains challenging. To systematically address this, Kahlon et al. (10) employed distance-based similarity measures in healthy adolescents, revealing that superior-inferior shank acceleration and medio-lateral shank angular velocity exhibit the highest robustness across varying speeds and walking conditions.

Building upon optimal signal selection, the CSAV method (39) demonstrates excellent agreement with optical motion capture (OMC) and force plates for toe-off detection, achieving high consistency in swing-phase kinematics. Extending these laboratory-validated approaches to free-living settings, Prigent et al. (71) recently demonstrated that a single foot-worn IMU enables reliable walking bout detection in unsupervised daily life, maintaining high sensitivity (>98%) and supporting real-world gait outcome extraction. This progression underscores a critical evolutionary trend: the transition from optimal signal identification and event extraction in standardized settings toward robust detection pipelines capable of handling the inherent complexity of real-world mobility (83, 84).

#### 3.12.2 Effectiveness of artificial intelligence algorithms

Machine learning (ML) algorithms offer inherent advantages in capturing nonlinear gait patterns and processing high-dimensional data; however, translating them into routine clinical practice faces three major barriers—applicability (clinical actionability), feasibility (resource constraints), and reliability (cross-population consistency) (85, 86). The ML-based leg-IMU abnormal gait classification algorithm (ML-LIGCA) (11) showcases superior classification performance, particularly in distinguishing knee vs. ankle injuries, surpassing previously reported accuracy ranges (76.3%–89.3%) while requiring fewer features (e.g., sagittal knee angle alone effectively differentiates normal from knee-injury gait). Similarly, the real-time lower-limb kinematic estimation framework integrating multiple linear regression with a Kalman filter (8) successfully bridges sensor minimalism and multi-joint kinematic richness, achieving 92.1% accuracy in classifying healthy adults and three distinct pathological gaits via a support vector machine classifier.

LSTM networks, through their gating mechanisms, enable effective learning from long temporal sequences, thereby generating more accurate and coherent kinematic estimations (87). The LSTM-based leg-IMU gait-phase estimation algorithm (LSTM-LIGPE) (44) achieved excellent validation accuracy (0.9977), although prediction errors occurred during turning or phases with substantial gait alterations. Beyond phase segmentation, Liang et al. (46) employed LSTM networks to model interlimb and intralimb synergies, significantly outperforming traditional principal component analysis in predicting contralateral joint angles. To further address the challenge of heterogeneous pathological gaits, Romijnders et al. (70) introduced a deep learning-based algorithm that detects precise gait events across multiple mobility-limiting diseases without requiring patient-specific calibration, demonstrating profound cross-disease generalizability.

Convolutional neural networks (CNNs) have also proven highly effective at filtering kinematic noise, as exemplified by Brand et al. (80), who extracted valid free-living gait sequences from wrist-worn IMUs despite complex arm-movement artifacts. Moreover, Kreuzer and Munz (45) developed a ConvLSTM network using an 11-sensor array that successfully classified five distinct gait phases with a mean accuracy of 92.29%, with feature importance analysis identifying thigh-mounted gyroscope data as most contributory. The weighted-average ensemble classification model (57) further demonstrated that multi-site IMU configurations combined with machine learning can discriminate early-stage Parkinson's disease from essential tremor with 75.8% accuracy.

Importantly, the heavy reliance on annotated datasets—a critical bottleneck for supervised AI models—is now being addressed by SSL paradigms. Recent frameworks such as ElderNet (82) and smartphone-based SSL applications (97) process vast amounts of unlabeled continuous data, drastically reducing annotation costs while maintaining high robustness across diverse unconstrained real-world scenarios, marking a pivotal frontier in AI-driven gait analysis.

#### 3.12.3 Effectiveness of algorithms in specific motor scenarios

For real-time applications, the detection of initial contact (IC) and terminal contact (TC)—along with its latency and robustness—is critical for accurate spatio-temporal parameter computation (88). To address this in running scenarios, Yang et al. (59) developed accelerometer- and gyroscope-based algorithms for IC/TC detection using foot-worn IMUs. Their system maintained high accuracy at moderate speeds but showed reduced performance at high speeds, influenced by forefoot strike patterns. Conversely, Siebers et al. (51) focused on stair ascent and descent and developed a novel multi-signal fusion algorithm that achieved 100% stride detection success in 35 healthy participants, outperforming existing comparative algorithms. This complementarity underscores the necessity of scenario-specific algorithm development for different motor tasks.

In the context of slow walking, the KF+ model algorithm (73)—tested on 10 healthy participants during treadmill walking—demonstrated high vertical displacement estimation accuracy. For stride length estimation, relative prediction errors remained below 10% at speeds ≥2 km/h with low standard deviation, providing an effective means for monitoring gait stability in older adults and patients with gait disorders. Furthermore, the low-cost outdoor IMU system (72), employing a Mahony complementary filter with Gauss–Newton optimization and ZUPT, achieved reliable outdoor gait tracking despite soft-tissue artifacts, demonstrating that classical sensor fusion frameworks remain relevant for embedded applications where computational constraints are stringent.

#### 3.12.4 Effectiveness of algorithms across different sensor placements and assessment objectives

Sensor placement fundamentally governs algorithmic performance. Rantalainen et al. (55) reported that waist-worn IMUs demonstrated good reliability and concurrent validity for estimating stride time variability using multiscale entropy, whereas ankle-worn IMUs showed only moderate reliability and poor validity, suggesting that entropy-based estimates are more suitable than event-detection-based methods for assessing gait variability with wearable sensors.

Regarding reference standards, optical motion capture (OMC) remains the established gold standard, although its high cost and time-consuming setup limit routine use (89, 90). Guimarães et al. (1) proposed an orientation-invariant foot-worn IMU method that effectively overcomes phe need for recise sensor alignment while achieving good agreement with OMC. Notably, as IMU reliability matures, Liu et al. (7) successfully utilized a seven-sensor multi-site IMU system as a clinical reference to validate a markerless AI vision system in pathological gaits, demonstrating that high-density IMUs can serve as independent ground truth where OMC is impractical.

Importantly, optimal sensor placement is highly task-dependent. Niswander et al. (9) systematically evaluated 11 anatomical locations during the timed-up-and-go test, revealing that standardized placements for straight-line walking are insufficient for complex clinical assessments. Beyond task-specific optimization, objectives such as comparing controlled capacity vs. free-living performance also dictate sensor strategies. Carcreff et al. (52) utilized a five-sensor setup to quantify the critical gap between higher laboratory capacity and actual daily-life performance in children with cerebral palsy.

The lower-back (waist) configuration has increasingly established itself as the robust standard for extracting longitudinal, real-world digital mobility outcomes (DMOs) across diverse cohorts. The Mobilise-D COPD gait analysis algorithm (74) demonstrated that walking speed, cadence, and stride length declinedsignificantly with increasing disease severity in 549 patients during 7-day continuous monitoring. Extending this to multi-pathological populations, Micó-Amigo et al. (76) validated lower-back algorithms across six cohorts, revealing that algorithm performance is highly cohort-specific and degrades significantly for slow walkers and short bouts. Mueller et al. (77) developed a waist-worn slow-gait estimation algorithm specifically calibrated for sarcopenic populations, accurately capturing real-world gait speeds below 0.5 m/s. Becker et al. (78) applied the Mobilise-D lower-back pipeline to 564 hip fracture patients across four recovery phases, capturing highly granular variations in DMOs that reflect longitudinal rehabilitation progress. Finally, Albites-Sanabria et al. (79) established normative benchmarks for healthy aging using lower back sensors in 200 community-dwelling older adults, revealing non-linear age- and sex-related trajectories of mobility decline.

When the primary objective shifts to maximizing long-term patient compliance, wrist-mounted IMUs (80)—paired with robust CNN algorithms—offer superior wearability despite inherent arm-movement noise.

#### 3.12.5 Effectiveness of algorithms for specific disease populations

Parkinson's disease (PD), the second most common neurodegenerative disorder, is projected to double in prevalence within 30 years, with growing evidence supporting physical therapy to alleviate motor symptoms (91, 92). The SDI-step algorithm (2)—validated through a defined processing pipeline—demonstrates small measurement errors in step length and time with good agreement with laboratory-grade systems, supporting PD rehabilitation therapy. Separately, Ullrich et al. (68) proposed a novel pipeline for detecting standardized gait tests during home monitoring, achieving an F1-score of 88.9% for test series detection and 94.0% for decomposition, with detected parameters showing strong correlation with manual annotations—providing reliable data support for clinical surveillance. Zhang et al. (3) further demonstrated that combined single- and dual-task gait parameters achieved an AUC of 0.924 for diagnosing early-stage PD, underscoring the diagnostic value of IMU-based gait metrics. Atrsaei et al. (75) utilized a lower-back IMU to assess fear of falling in PD patients, revealing that combining laboratory capacity metrics with daily-activity performance data significantly improved classification accuracy (AUC = 0.75). Ferrari et al. (53) employed a three-sensor multi-site configuration to objectively quantify improvements in gait apraxia in patients with idiopathic normal pressure hydrocephalus following clinical interventions, demonstrating significantly higher sensitivity than traditional stopwatch assessments.

Stroke remains a leading cause of disability, with post-stroke motor dysfunction severely affecting daily living and quality of life (93, 94). FES has been employed to enhance physical performance and counteract muscle atrophy in neurological disorders (95, 96). Feuvrier et al. (61) used the Martin and Salaun algorithm and demonstrated good agreement between IMU and OMC measurements in post-stroke foot-drop patients, strongly supporting IMU-FES integration for adaptive stimulation parameter adjustment. From a complementary perspective, Schicketmueller et al. (64) developed an algorithm for triggering FES during robot-assisted gait training, achieving high gait event detection accuracy in both the Lyra and Lokomat systems (detection rates > 80% and >90%, respectively), laying the groundwork for precise FES-triggered rehabilitation interventions. It is important to note that individual responses to FES vary due to factors such as muscle fatigue, time-varying spasticity, and sensitivity to electrode placement (96), underscoring the rationale for subsequent IMU-FES integration to develop adaptive stimulation strategies.

Synthesizing the methodological profiles presented in Table 3, three cross-cutting insights emerge. First, validation paradigms remain heavily skewed toward laboratory settings: of the 33 algorithm entries profiled, the majority (*n* = 22) were validated exclusively in controlled environments, with only 11 incorporating free-living or unsupervised monitoring components—a gap that challenges translation to real-world clinical practice. Second, there is a positive association between sample size and reported validation robustness: the largest-scale studies [e.g., Mobilise-D COPD cohort (74), *n* = 568; RUSH MAP wrist validation (82), *n* = 819] consistently reported stronger cross-cohort generalizability, whereas smaller studies (*n* < 30) frequently reported excellent within-sample accuracy but acknowledged limited external validity. Third, the relationship between sensor placement and algorithm type is non-random: foot-mounted configurations more frequently employed deep learning for event detection (70), while lower-back placements predominantly utilized rule-based or standardized pipelines (74, 76–79), reflecting distinct signal characteristics and computational constraints. These observations underscore that methodological choices—sensor placement, algorithm selection, and validation design—are deeply intertwined and that reported performance must be interpreted in light of the specific validation context, sample characteristics, and environmental conditions under which each algorithm was tested.

#### 3.12.6 Emerging direction: self-supervised learning for gait event detection using smartphone IMUs

In recent years, SSL-based gait event detection using smartphone inertial measurement units has emerged as a transformative approach to overcoming the limitations of conventional methods (97). Leveraging smartphones as ubiquitous IMU carriers, this approach eliminates the need for additional dedicated wearable sensors while supporting versatile wearing scenarios without fixed body placement constraints. Its core advantage lies in contrastive pretraining on massive unlabeled data, requiring only minimal labeled data for downstream fine-tuning. Integrated with sensor-aware augmentation strategies, it maintains robustness across cross-device scenarios and varying sampling rates. As a critical complement to the “fixed-placement IMU methods” reviewed in this article—particularly the wrist-worn SSL framework (82)—this paradigm addresses key limitations of traditional approaches and facilitates convenient data collection in sports and community settings where dedicated IMUs are impractical. This provides a feasible pathway for large-scale applications such as athlete load monitoring and community health screening, substantially broadening the practical deployment potential of gait analysis technologies.

## 4 Discussion

This systematic review synthesizes contemporary gait analysis screening methodologies utilizing inertial measurement units (IMUs), systematically categorized by anatomical sensor placement (i.e., foot, lower limb, chest, lower back/waist, wrist, and multi-site configurations). The synthesized evidence suggests that a universally optimal algorithm or a singular sensor configuration is unlikely to exist based on the current literature. Rather, the efficacy and reliability of any given methodological pipeline appear to depend on a multidimensional interplay of specific clinical assessment objectives, heterogeneous pathological profiles, environmental contexts (controlled laboratory vs. unconstrained free-living), and computational resource constraints. Consequently, the primary contribution of this review lies in mapping these complex context-method dependencies, ultimately providing an evidence-based framework to guide researchers and clinicians in the informed selection of scenario-specific gait analysis solutions.

### 4.1 Core comparison of algorithm types and sensor configurations

Combining the extracted data, key insights into the evolutionary trajectory of IMU-based gait analysis methods emerge. From an algorithmic perspective, traditional rule-based approaches [e.g., CSAV (39), Martin and Salaun (61)] feature low computational complexity, high stability, and reliable consistency with reference standards like optical motion capture in controlled settings, making them potentially suitable for real-time clinical feedback or low-power embedded devices. In contrast, supervised learning models [e.g., ML-LIGCA (11), LSTM-LIGPE (44)] demand more computational resources but show promise in classifying specific pathological gait patterns and accommodating individual variability. Pushing these boundaries further, advanced deep learning architectures [such as CNNs (80)] have demonstrated capabilities in generalizing across heterogeneous diseases without patient-specific calibration and robustly handling severe kinematic noise (70). Furthermore, the emerging paradigm of SSL may address the critical bottleneck of relying on massive, heavily annotated datasets, marking a potential shift toward scalable, unsupervised analysis in free-living environments (82, 97). However, it is important to note that many of these deep learning approaches have been validated on relatively small or homogeneous datasets; their generalizability to broader, more diverse populations remains to be investigated.

Regarding sensor configurations, a distinct tradeoff exists between kinematic comprehensiveness and longitudinal wearability. Single-sensor setups on the foot or chest traditionally balance wearability with basic parameter accuracy (1, 2, 73). However, lower-back (waist) placements have emerged as a well-established standard for extracting longitudinal, real-world digital mobility outcomes (DMOs) across diverse, slow-walking, and frail cohorts, particularly through the standardized Mobilise-D pipeline (74, 76–79). When maximizing long-term patient compliance is the primary clinical objective, wrist-mounted sensors offer favorable wearability, although they require highly complex algorithms to isolate gait from extraneous arm movements (80, 82). Conversely, multi-site configurations provide the most comprehensive kinematic capture (45, 46, 52, 53, 57, 72). While imposing higher patient burden, they appear particularly valuable for evaluating complex postural transitions (e.g., turning, stair climbing) and precisely quantifying the discrepancy between supervised laboratory capacity and actual daily-life performance in complex neurological populations (52, 53). Nevertheless, the additional value of multi-site configurations over well-optimized single-sensor setups in routine clinical applications remains to be systematically quantified.

### 4.2 Correlation between method performance and applicable scenarios

The analysis reveals a notable complementarity among different sensor wear locations, their corresponding algorithmic architectures, and specific clinical scenarios. The foot-mounted IMU most directly captures key micro-events in the gait cycle, such as heel strike and toe-off. It appears to achieve high accuracy in measuring spatio-temporal parameters in controlled settings, making it potentially suitable for assessing neurological disorders that require precise event detection (e.g., Parkinson's disease and post-stroke foot drop) (1, 2, 61, 68), as well as extracting unconstrained real-world walking bouts (70, 71). The leg-mounted configuration excels at capturing overall lower-limb kinematic patterns. It performs well in identifying joint-specific impairments (e.g., knee and ankle abnormalities) and executing fine-grained gait phase estimation, which is largely attributable to the ability of machine learning models to analyze localized motion patterns of the thigh and shank (8, 10, 11, 39, 44).

Importantly, achieving high-fidelity joint kinematics is highly sensitive to the exact anatomical placement. As demonstrated by Niswander et al., who systematically evaluated 11 different lower-body IMU locations against optical motion capture during TUG tests, both mean bias and RMSE fluctuate significantly across target joints and functional movements (9). This finding suggests that before deploying complex analytical models, optimizing evidence-based hardware placement is an important consideration for minimizing fundamental measurement errors.

In contrast, the lower-back (or waist/chest) configuration—while offering slightly lower precision in micro-level spatio-temporal parameters—provides notable advantages in wearability, overall gait variability evaluation, and macro-level stability assessment (55, 73). Building upon this foundation, it has become a commonly adopted standard for long-term, unconstrained digital mobility outcome (DMO) extraction across diverse cohorts (e.g., COPD and hip fracture recovery) (74, 78) and appears particularly useful for tailored slow-gait estimation in frail, sarcopenic populations (77). The Mobilise-D healthy aging benchmark pipeline (79) further extends this utility by establishing normative real-world gait references, potentially enabling clinicians to distinguish natural age-related changes from pathological decline. Furthermore, when maximizing longitudinal patient compliance is the overriding clinical objective, wrist-mounted sensors offer superior feasibility when advanced AI algorithms (e.g., CNNs and self-supervised learning) are deployed to filter out the inherent kinematic noise of complex arm movements (80, 82). Finally, multi-site configurations, while imposing a higher setup burden, appear particularly valuable for capturing complex motor transitions (e.g., turning and sit-to-stand) and accurately quantifying the discrepancy between supervised clinical capacity and unsupervised daily performance in complex neurological conditions (52, 53, 57).

### 4.3 Tradeoff between algorithmic complexity and clinical feasibility

The computational complexity of the various algorithms was systematically compared in this study (Table 2). A key finding is that high accuracy often comes at the cost of high computational expense. For instance, while LSTM-based models support complex gait patterns and enable end-to-end automated analysis, their high computational requirements and annotated data limit their feasibility in resource-constrained environments or real-time applications (44–46). In contrast, lightweight methods such as the CSAV (39), Martin and Salaun (61), and OI-STGA (1) algorithms are highly computationally efficient due to their focused functionality, making them more suitable for embedded devices or clinical scenarios requiring rapid feedback. The low-cost outdoor IMU system (72) further exemplifies how classical sensor fusion frameworks can achieve reliable outdoor gait tracking without relying on costly commercial algorithms.

However, as clinical objectives increasingly shift toward unconstrained, free-living environments, the requirements for algorithmic robustness inevitably escalate. Advanced deep learning architectures, such as CNNs (80), offer superior noise-filtering capabilities for challenging sensor placements (e.g., the wrist) but intrinsically amplify computational demands. To counterbalance this complexity and enhance clinical feasibility, recent deep learning frameworks have shown promise by achieving high cross-disease accuracy without the need for patient-specific calibration (70). Furthermore, the profound challenge of acquiring massive, manually annotated datasets—a primary feasibility bottleneck for supervised models like LSTMs—is now being actively addressed by the emerging SSL paradigm. By directly leveraging vast amounts of unannotated continuous data from wearables and smartphones (82, 97), SSL may drastically reduce data engineering costs and implementation barriers while maintaining the high performance of complex neural networks. Nevertheless, the clinical validation of SSL-based approaches is still in its early stages, and their robustness across diverse patient populations and real-world conditions awaits confirmation.

This indicates that a careful tradeoff must be made between algorithmic performance, computational efficiency, and implementation cost when selecting a method. An important future direction involves developing lightweight intelligent algorithms that maintain acceptable accuracy while reducing computational burden, alongside the integration of robust SSL pipelines to maximize large-scale clinical feasibility in real-world settings.

### 4.4 Challenges and opportunities in clinical translation

Despite the promising prospects of IMU technology, its standardization and widespread translation into clinical practice face significant challenges. First, the lack of unified benchmark datasets and validation protocols hinders direct comparison of results across different studies (Table 3). Fortunately, emerging initiatives are now establishing standardized digital mobility outcomes (DMOs) and normative baselines across cohorts (76, 79) to facilitate unified clinical adoption.

Second, as most existing algorithms are developed for specific populations or scenarios (e.g., healthy adults, level walking), their generalizability and robustness in patients with multiple comorbidities or in complex real-world environments (e.g., crowded spaces, uneven terrain) remain to be validated (83, 84). Since universal models often degrade in vulnerable populations or during complex postural transitions (53, 75, 77), future research should prioritize disease- and scenario-specific algorithmic tailoring.

Third, the clinical translation of IMU-based gait analysis must overcome the gap between laboratory-validated accuracy and real-world applicability. While reference standards such as optical motion capture (89, 90) and pressure insoles provide high-fidelity validation, translating these research-grade accuracies into routine clinical workflows requires addressing practical barriers including device cost, data interpretation complexity, and integration with existing electronic health record systems.

**Figure 4:**
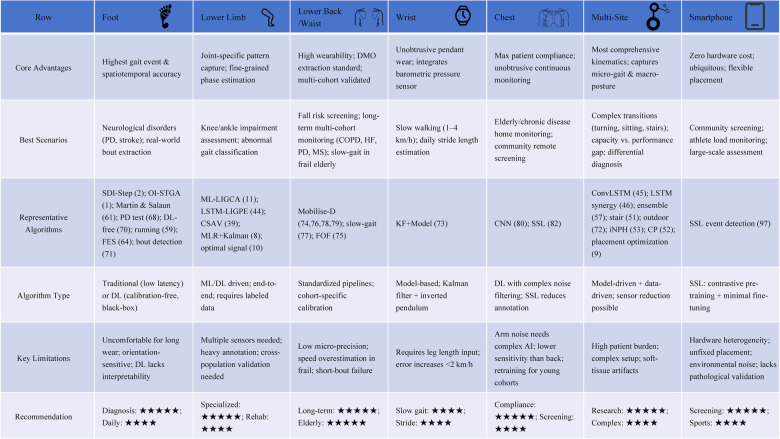
Practical decision guide for IMU-based gait analysis.

Furthermore, integrating laboratory-validated, high-accuracy algorithms into clinical workflows or patients' daily monitoring regimes, and subsequently generating intuitive, actionable reports, is important for realizing their clinical value. Bridging this gap requires addressing the discrepancy between clinical “capacity” and daily “performance” (52). Utilizing high-compliance placements (e.g., the wrist) (80) alongside SSL for unannotated data (82, 97) offers a practical pathway forward, enabling scalable, continuous monitoring that captures meaningful real-world mobility outcomes. Figure 4 provides a comprehensive decision guide to assist researchers and clinicians in navigating these complex tradeoffs when selecting IMU-based gait analysis solutions for specific applications.

#### 4.4.1 Limitations of the current review

Despite the rigorous systematic approach adopted, several limitations of this review should be acknowledged. First, there is significant methodological heterogeneity across the 30 included studies. Variations in sensor locations (e.g., wrist, waist, shank, foot), algorithmic architectures, and sampling frequencies, combined with the diverse target pathological cohorts, severely limit the direct comparability between different validation protocols. Consequently, conducting a quantitative meta-analysis was not feasible, and this study was limited to a qualitative narrative synthesis of current evidence. Second, despite conducting a comprehensive search across four major databases, potential publication bias cannot be entirely ruled out, as algorithms yielding negative, null, or suboptimal accuracy results are historically less likely to be published. Furthermore, although both English and Chinese databases were searched to ensure broad coverage, potential language bias may still exist, as relevant literature published in other languages may have been inadvertently omitted. Finally, it is important to note that the scope of this review was strictly focused on original peer-reviewed algorithm development. While this ensures methodological purity, the exclusion of secondary systematic reviews and clinical-only observational studies means that broader clinical perspectives—which might be found in such documents—were not the primary focus of this synthesis.

### 4.5 Practical decision guide for IMU-based gait analysis

To facilitate the rapid selection of suitable IMU-based gait analysis protocols according to application requirements in practical scenarios including clinical diagnosis and treatment as well as sports health monitoring, the present study developed a practical application decision guide for IMU-based gait analysis by synthesizing the technical characteristics, scenario adaptability, and algorithmic performance of IMU deployments at different body locations as discussed above, with details presented in Figure 4. This guide is intended to provide preliminary orientation rather than definitive prescriptions; clinicians and researchers should consider their specific context, available resources, and patient population when making final decisions.

The matrix compares seven sensor placements across six dimensions: core advantages, best scenarios, representative algorithms, algorithm type, key limitations, and recommendations. This guide explicitly distinguishes lower-back/waist placements (optimized for longitudinal DMO extraction) from chest/upper-back placements (suited for stride length estimation). Star ratings (★) indicate relative strengths. DMO: digital mobility outcome; SSL: self-supervised learning; PD: Parkinson's disease; COPD: chronic obstructive pulmonary disease; CHF: congestive heart failure; MS: multiple sclerosis; iNPH: idiopathic normal pressure hydrocephalus; CP: cerebral palsy; DL: deep learning; ML: machine learning; FES: functional electrical stimulation. Algorithm references are in parentheses.

### 4.6 Future perspectives

Based on the findings of this systematic review, five core research directions for inertial measurement unit (IMU)-based gait analysis are proposed.

First, priority should be given to the development of adaptive and personalized intelligent algorithms. Such algorithms should be able to automatically adjust their parameters according to individual anatomical variations, walking habits, and actual motor task scenarios. By moving beyond generic models, these tailored deep learning architectures may improve generalization ability and robustness across diverse pathological populations and complex environments, addressing the limitation of single adaptability in existing algorithms. This is particularly important given the cohort-specific performance degradation observed in current models (76, 77).

Second, advance the research and development of multimodal data fusion technology as a core approach to break through the performance limitations of single IMU systems. Combining IMUs with other physiological sensing modalities—including low-cost photoelectric sensors and surface electromyography sensors—enables the synchronous acquisition of human kinematic characteristics and physiological signal information, thus potentially enabling a more accurate and comprehensive multi-dimensional assessment of motor function.

Third, explore the in-depth integrated application of IMUs built into smartphones using SSL technology, which opens a novel pathway for the large-scale, low-cost popularization of gait analysis techniques (97). Future research should focus on optimizing smartphone-based gait analysis algorithms to address key issues, such as device hardware heterogeneity, variable wearing positions, and interference from complex daily environments, thereby improving the detection robustness of algorithms. Leveraging the high penetration rate of smartphones and the capability of SSL to process massive unannotated data, this technology can be practically applied in scenarios including community-level health screening, remote gait monitoring for patients with neurological disorders, and long-term exercise load management for athletes.

Fourth, construct a standardized, open publicly available benchmark dataset, which is an important prerequisite for promoting the standardized and high-quality development of this field. Building upon current large-scale initiatives (e.g., Mobilise-D) (76, 78, 79), the dataset should comprehensively cover healthy populations, various populations with pathological gait, and diverse real-world movement scenarios such as level walking, stair ascent and descent, and walking on uneven terrain (83, 84). It would provide a unified basis for the fair comparison and reproducibility verification of different algorithms, thereby potentially accelerating the iteration and innovation of IMU-based gait analysis technologies.

Finally, future research should place clinical utility and user experience at the core. This requires promoting in-depth collaboration among researchers, clinicians, and patients, who will jointly design technical solutions with distinct clinical value, good user-friendliness (e.g., high-compliance wrist or waist placements) (80, 82), and seamless integration into existing healthcare systems. Ultimately, this may facilitate the large-scale practical application of IMU-based gait analysis technology, moving it from laboratory research to clinical diagnosis and treatment, daily health monitoring, and other real-world scenarios.

## 5 Conclusion

In conclusion, this systematic review suggests that IMU-based gait analysis has progressively evolved from a constrained laboratory tool toward a promising paradigm for continuous, real-world mobility assessment. The available evidence indicates that no universally optimal algorithm or singular sensor placement exists. Instead, achieving clinically meaningful results requires careful consideration of the tradeoffs between biomechanical comprehensiveness, analytical accuracy, longitudinal patient compliance, and the specific constraints of each application scenario. As the field continues to transition toward unconstrained free-living environments, integrating context-aware sensor configurations into advanced, annotation-efficient artificial intelligence approaches may play an important role in translating complex kinematic data into standardized, clinically actionable insights. However, the strength of these conclusions is moderated by the methodological limitations of the primary evidence base, including small sample sizes, population-specific validation, and predominantly laboratory-based testing environments. Future large-scale, multi-center validation studies conducted in real-world settings are needed to confirm the generalizability of the findings summarized in this review.
