# Supplementary material for: IMU-based gait analysis methods: a systematic review of techniques for different body locations
Source: Front Sports Act Living. 2026 Jul 31;8:1854615. doi: 10.3389/fspor.2026.1854615 (PMC13474989; doi:10.3389/fspor.2026.1854615)
Supplement: Supplementary file 1 [file Supplementaryfile1.docx]

Pubmed

(IMU OR "Inertial Measurement Unit" OR "Inertial Sensor" OR "Wearable Sensor") AND (Gait OR "Gait Analysis" OR "Mobility Assessment" OR "Walking Speed") Filters: from 2019 - 2025

Web of Science

Refine results for (IMU OR "Inertial Measurement Unit" OR "Inertial Sensor" OR "Wearable Sensor") AND (Gait OR "Gait Analysis" OR "Mobility Assessment" OR "Walking Speed") and 2025 or 2024 or 2023 or 2022 or 2021 or 2020 or 2019 (Publication Years)

CNKI

("IMU" OR "惯性测量单元" OR "惯性传感器" OR "可穿戴传感器")) AND (SU=("步态" OR "步态分析" OR "行动能力评估" OR "步行速度")) AND (Date BETWEEN '2019-01-01' AND '2025-12-31')

Wanfang

(主题:("IMU" OR "惯性测量单元" OR "惯性传感器" OR "可穿戴传感器")) AND (主题:("步态" OR "步态分析" OR "行动能力评估" OR "步行速度")) AND Date:2019-2025
